# Targeting the Lysosomal Degradation of Rab22a‐NeoF1 Fusion Protein for Osteosarcoma Lung Metastasis

**DOI:** 10.1002/advs.202205483

**Published:** 2022-12-18

**Authors:** Cuiling Zeng, Li Zhong, Wenqiang Liu, Yu Zhang, Xinhao Yu, Xin Wang, Ruhua Zhang, Tiebang Kang, Dan Liao

**Affiliations:** ^1^ State Key Laboratory of Oncology in South China Sun Yat‐sen University Cancer Center Collaborative Innovation Center for Cancer Medicine Guangzhou 510060 China; ^2^ Center of Digestive Diseases The Seventh Affiliated Hospital, Sun Yat‐sen University Shenzhen 518107 China; ^3^ Scientific Research Center The Seventh Affiliated Hospital, Sun Yat‐sen University Shenzhen China; ^4^ Department of Oncology The Fifth Affiliated Hospital, Sun Yat‐sen University Zhuhai 519000 China

**Keywords:** degradation, fusion protein, metastasis, osteosarcoma, phosphorylation

## Abstract

Rab22a‐NeoF fusion protein has recently been reported as a promising target for osteosarcoma lung metastasis. However, how this fusion protein is regulated in cells remains unknown. Here, using multiple screenings, it is reported that Rab22a‐NeoF1 fusion protein is degraded by an E3 ligase STUB1 via the autophagy receptor NDP52‐mediated lysosome pathway, which is facilitated by PINK1 kinase. Mechanistically, STUB1 catalyzes the K63‐linked ubiquitin chains on lysine112 of Rab22a‐NeoF1, which is responsible for the binding of Rab22a‐NeoF1 to NDP52, resulting in lysosomal degradation of Rab22a‐NeoF1. PINK1 is able to phosphorylate Rab22a‐NeoF1 at serine120, which promotes ubiquitination and degradation of Rab22a‐NeoF1. Consistently, by upregulating PINK1, Sorafenib and Regorafenib can inhibit osteosarcoma lung metastasis induced by Rab22a‐NeoF1. These findings reveal that the lysosomal degradation of Rab22a‐NeoF1 fusion protein is targetable for osteosarcoma lung metastasis, proposing that Sorafenib and Regorafenib may benefit cancer patients who are positive for the *RAB22A*‐NeoF1 fusion gene.

## Graphical abstract

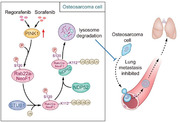

## 1 Introduction

Osteosarcoma, one of the most common malignant bone cancers with high peak incidence in adolescents,^[^ ^1^ ^]^ has up to a 70% survival rate after local surgery combined with neoadjuvant multidrug chemotherapy.^[^ ^2^ ^]^ However, the 5 years overall survival rate has still remained at only 20% over the past 30 years because 15–30% of patients already present lung metastasis at diagnosis.^[^ ^3^ ^]^ Redundancy in growth signals and high heterogeneity results in no effective molecular therapeutic targets in osteosarcoma.^[^ ^4^ ^]^ Our group has just identified new fusion genes termed *RAB22A*‐NeoFs, the incidence rate for these fusion genes was 5.4% (2 out 37) in osteosarcoma patients with metastases, whereas this type of fusion genes was not detected in 60 osteosarcoma patients without metastasis.^[^ ^5^ ^]^ RAB22A‐NeoF1 fusion gene is derived from chromosomal translocations that juxtapose amino acids 1–38 of Rab22a with inverted intron sequences of *DOK5*. This fusion gene can encode Rab22a‐NeoF1 fusion protein to drive osteosarcoma lung metastasis in multiple ways and may be a promising target for patients with osteosarcoma metastases.^[^ ^5^, ^6^ ^]^ However, how this fusion protein is regulated in cells remains unexplored.

Proteostasis, the maintenance of a healthy proteome,^[^ ^7^ ^]^ protects against the detrimental consequences of unfolded, misfolded, or damaged proteins that severely disturb cellular functions and induce malignant tumors and immunological diseases. The ubiquitin‐proteasome system (UPS) and the autophagy‐lysosome pathway (ALP) are two major protein degradation systems by the K48‐linked ubiquitin chains and the K63‐linked ubiquitin chains, respectively.^[^ ^8^ ^]^ The topology of ubiquitin chains is often associated with different biological roles. The K48‐linked ubiquitin chains are usually associated with the targeting of proteins to the proteosome.^[^ ^9^ ^]^ In contrast, K63 linked chains have been involved in DNA repair, internalization of plasma membrane proteins, protein sorting to multivesicular bodies, and protein and/or subcellular organelles degradation in the lysosome by macroautophagy.^[^ ^10^ ^]^ ALP can be either selective or nonselective in degrading substrates by unique double‐membrane autophagosome that fuses with lysosome. In the case of selective autophagy, cargo is recognized by specific receptors.^[^ ^11^ ^]^ Notably, Rab22a‐NeoF1 fusion protein could be stabilized in cells treated with proteasome inhibitor MG132 plus lysosome inhibitor BafA1.^[^ ^5^ ^]^ This indicated that Rab22a‐NeoF1 fusion protein may be degraded by proteasome and/or lysosome, potentially providing an excellent opportunity to target this fusion protein by enhancing its degradation.

In this report, using multiple screenings, we found that Rab22a‐NeoF1 fusion protein is polyubiquitinated at lysine112 by an E3 ligase STUB1 to be recognized by autophagy receptor NDP52 and subsequently degraded by lysosome. This lysosomal degradation of Rab22a‐NeoF1 could be enhanced by the phosphorylation of serine120 by PINK1 kinase. Two targeted drugs, Sorafenib and Regorafenib, could upregulate PINK1 to diminish osteosarcoma lung metastasis induced by Rab22a‐NeoF1.

## 2 Results

### 2.1 Flow Cytometry‐Based CRISPR‐Cas9 Screening Identifies STUB1 as an E3 Ligase Targeting Rab22a‐NeoF1 Fusion Protein

**Figure 1:**
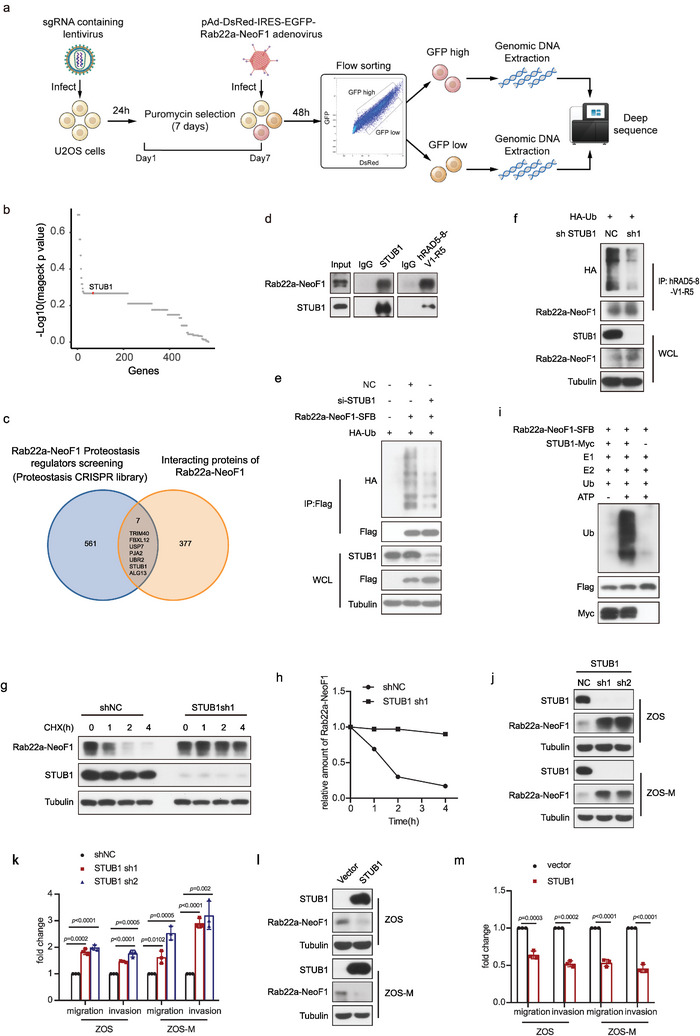
Flow cytometry‐based CRISPR‐Cas9 knockout library screening reveals STUB1 as an E3 ligase targeting Rab22a‐NeoF1 fusion protein in osteosarcoma. a) Schematic of CRISPR‐Cas9 knockout library screening pipeline and the gating strategy used in the screen. b) Scatterplot showing average log10 fold changes in mageck *p* value of sgRNA abundance in the GFP ^high^‐sorted population. sgRNAs targeting STUB1 are highlighted in red. Each dot represents an average of log10 fold changes in mageck *p* values for ten independent sgRNAs per gene. c) Venn diagram indicates overlap of genes between the enrichment of sgRNAs targeting Rab22a‐NeoF1 in the CRISPR‐Cas9 knockout library screening and the interactome of Rab22a‐NeoF1 fusion protein by mass spectrometry. d) The co‐IPs were performed using ZOS‐M cells with IgG, hAb RAD5‐8, or anti‐STUB1 at their endogenous levels, and were analyzed by western blot. The experiments were repeated three times independently with similar results. e) Transfection of siRNAs targeting STUB1 or negative control (NC) in the 293T cells for 24 h and then the indicated plasmids were reinfected into these cells for 36 h. Cells were lysed and immunoprecipitated using anti‐FLAG beads followed by western blot analysis. The experiments were repeated three times independently with similar results. f) ZOS cells with knockdown of STUB1 by shRNAs or NC as indicated were transfected with HA‐Ubiquitin (HA‐Ub) for 48 h and then were lysed and immunoprecipitated using hRAD5‐8‐V1‐R5 antibody followed by western blot analysis. The experiments were repeated three times independently with similar results. g,h) ZOS cells with knockdown of STUB1 by shRNAs or shNC as indicated were treated with g) cycloheximide (CHX: 20 µg mL^−1^) for the indicated time and subjected to western blotting analysis. h) Quantitation of the Rab22a‐NeoF1 protein levels in (g). The experiments were repeated three times independently with similar results. i) The indicated Rab22a‐NeoF1‐SFB and STUB1‐Myc protein were affinity‐isolated using streptavidin‐conjugated beads and anti‐Myc affinity gel, respectively, from lysates of HEK293T cells transfected with SFB‐tagged Rab22a‐NeoF1 or MYC‐tagged STUB1 for 48 h, and then were subjected to the in vitro ubiquitination assay. The experiments were repeated three times independently with similar results. j,l) The indicated stable ZOS or ZOS‐M cells were analyzed by western blot. The experiments were repeated three times independently with similar results. k,m) Quantification analyses of migration and invasion assays using the indicated stable ZOS or ZOS‐M cells. The depicted results are the averages of at least three independent experiments. The data are presented as the mean ± SD. A two‐sided unpaired student's *t*‐test was performed, and *p* values are shown.

Our group has recently reported that Rab22a‐NeoF1 fusion protein may be a promising target for osteosarcoma metastasis.^[^ ^5^, ^6^ ^]^ We have previously showed that Rab22a‐NeoF1 fusion protein could be stabilized in cells treated with proteasome inhibitor MG132 plus lysosome inhibitor BafA1 (extended data Fig. 2a of ref. [7]), indicating that Rab22a‐NeoF1 fusion protein may be degraded by proteasome and/or lysosome. To explore how Rab22a‐NeoF1 fusion protein is degraded, we generated U2OS cells expressing DsRed‐IRES‐EGFP‐Rab22a‐NeoF1. Because concurrent examination of the protein levels for GFP‐Rab22a‐NeoF1 and DsRed control allows us to identify the regulators for Rab22a‐NeoF1 fusion protein stability by a flow cytometry‐based screen, we have designed this assay as the protein stability regulators screening assay (Pro‐SRSA).^[^ ^12^ ^]^ The U2OS cells were infected with a CRISPR‐Cas9 knockout library, including sgRNAs targeting all E1, E2, and E3 ligases (709 genes); deubiquitinases (111 genes); (de)methytranferases (88 genes); and (de)acetyllationases (35 genes). After being passaged for 7 days, these cells were infected with DsRed‐IRES‐EGFP‐Rab22a‐NeoF1 adenovirus for 48 h, and then GFP^low^ and GFP^high^ populations were sorted to be deep sequencing (**Figure** **1**a), and we had an enrichment of sgRNAs targeting Rab22a‐NeoF1 in the GFP^high^ subpopulation compared with the GFP^low^ subpopulation (Figure 1b and Table S4, Supporting Information). In combination with the Rab22a‐NeoF1 fusion protein interactome we reported previously,^[^ ^5^ ^]^ seven genes (*STUB1*, *PJA2*, *UBR2*, *TRIM40*, *FBXL12*, *USP7*, and *ALG13*) were found to be overlapped in these two analyses, indicating that they may potentially regulate Rab22a‐NeoF1 fusion protein stability (Figure 1c). The protein level of Rab22a‐NeoF1 was stabilized by siRNA‐knockdown of *STUB1, PJA2*, or *ALG13* but not *UBR2*, *TRIM40*, *FBXL12*, or *USP7* (Figure S1a, Supporting Information), and the efficient siRNA‐knockdown of these genes was individually verified by quantitative polymerase chain reaction (Figure S1b, Supporting Information). Because ALG13 is neither an E3 ligase nor a deubiquitinase, and because ectopic STUB1 but not PJA2 could decrease ectopic Rab22a‐NeoF1 protein (Figure S1c, Supporting Information), we surmised that *STUB1* encodes for STIP1 homology and U‐box containing protein 1, an E3 ligase that may regulate Rab22a‐NeoF1 fusion protein stability.

Indeed, using Rab22a‐NeoF1 stability reporter, knockout of STUB1 stabilized Rab22a‐NeoF1 fusion protein (Figure S1d, Supporting Information), and ectopic STUB1 could bind and polyubiquitinate ectopic Rab22a‐NeoF1 but not ectopic Rab22a (Figure S1e–g, Supporting Information). The tetratricopeptide repeat (TPR) domain of STUB1 was responsible for its binding to Rab22a‐NeoF1 (Figure S1h,i, Supporting Information) and the K30A mutant within the TPR domain of STUB1 but not the H260Q mutant that lacks E3 ligase activity, completely abolishing its interaction with Rab22a‐NeoF1 (Figure S1j, Supporting Information). Neither K30A mutant nor H260Q mutant were able to polyubiquitinate Rab22a‐NeoF1 (Figure S1k, Supporting Information). Furthermore, the interaction between STUB1 and Rab22a‐NeoF1 was clearly detectable in ZOS‐M cells at their endogenous levels (Figure 1d). Knockdown of STUB1 by shRNA or siRNA resulted in polyubiquitination inhibition and half‐life prolongation of either ectopic or endogenous Rab22a‐NeoF1 (Figure 1e–h and Figure S1l,m, Supporting Information). Consistently, STUB1 could ubiquitinate Rab22a‐NeoF1 using the in vitro ubiquitination assay (Figure 1i). Knocking down of STUB1 by shRNAs could increase endogenous Rab22a‐NeoF1 fusion protein levels as well as cell migration and invasion in both ZOS and ZOS‐M cells (Figure 1j,k, Supporting Information), whereas overexpression of STUB1 had opposite effects in both ZOS and ZOS‐M cells (Figure 1l,m, Supporting Information). Collectively, we demonstrated that STUB1 is an E3 ligase that regulates Rab22a‐NeoF1 fusion protein stability in cells.

### 2.2 K112 is Critical for the STUB1‐Mediated Degradation of Rab22a‐NeoF1

**Figure 2:**
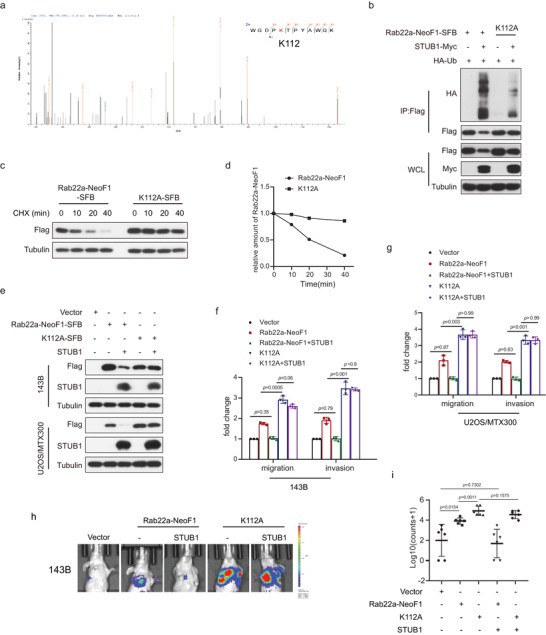
K112 is critical for the STUB1‐mediated degradation of Rab22a‐NeoF1 fusion protein. a) Lysine112 (K112) was ubiquitinated by mass spectrometry analysis of the immunoprecipitation complexes using anti‐FLAG beads from U2OS cells stably expressing Rab22a‐NeoF1‐SFB and STUB1‐Myc. b) 293T cells co‐transfected with the indicated plasmids for 48 h were lysed and immunoprecipitated using anti‐FLAG beads followed by western blot analysis. The experiments were repeated three times independently with similar results. c,d) UO2S cells stably expressing Rab2a‐NeoF1's wild type or its K112A mutant as indicated were treated with c) cycloheximide (CHX: 20 µg mL^−1^) for the indicated time and were subjected to western blotting analysis. d) Quantitation of the Rab22a‐NeoF1 protein levels in (c). The experiments were repeated three times independently with similar results. e) 143B cells and UO2S/MTX300 cells stably expressing Rab22a‐NeoF1's wild type or its K112A mutant were stably transfected with vector or STUB1 as indicated and then were analyzed by western blotting. The experiments were repeated three times independently with similar results. f,g) Quantification analyses of migration and invasion assays using the indicated stable cell lines shown in (e). The depicted results are the averages of at least three independent experiments. The data are presented as the mean ± SD. A two‐sided unpaired student's *t*‐test was performed and *p* values are shown. h,i) The orthotropic osteosarcoma metastasis model in vivo using the indicated stable cell lines are shown in (e) (*n* = 6 biologically independent animals). h) Representative images of mice. i) Quantification analyses of images.

Next, we explored how STUB1 regulates Rab22a‐NeoF1 fusion protein. Ubiquitin has seven lysine residues, and each lysine‐linked ubiquitin chain (K6, K11, K27, K29, K33, K48, and K63) tagged with HA was generated by mutating other lysine residues into arginine residues. As shown in Figure S2a in the Supporting Information, only the K63‐linked ubiquitin chain (K63) was similar to wide‐type ubiquitin when each HA‐ubiquitin was co‐transfected with Rab22a‐NeoF1‐SFB and STUB1‐Myc, indicating that the polyubiquitination of Rab22a‐NeoF1 fusion protein by STUB1 was the K63‐linked ubiquitin chain. Lysine112 (K112) was the only ubiquitination site revealed by mass spectrometry (**Figure** **2**a). Indeed, when all 11 lysine residues within Rab22a‐NeoF1 were mutated individually into alanine (A), only K112A mutant of Rab22a‐NeoF1 fusion protein was resistant to degradation by STUB1 (Figure S2b, Supporting Information). Consistently, polyubiquitination and half‐life of Rab22a‐NeoF1 K112A mutant protein were diminished and prolonged compared to its wild type, respectively (Figure 2b–d, Supporting Information). Migration and invasion of both 143B and U2OS/MTX300 cells stably expressing the K112A mutant of Rab22a‐NeoF1 were enhanced compared to those stably expressing Rab22a‐NeoF1 (Figure 2e–g, Supporting Information). The enhancement of both migration and invasion were abolished by ectopic STUB1 in both 143B and U2OS/MTX300 stably expressing Rab22a‐NeoF1 but not in those stably expressing the K112A mutant of Rab22a‐NeoF1 (Figure 2e–g, Supporting Information). More importantly, the orthotopic osteosarcoma lung metastasis model was explored in vivo using 143B‐luc cells, and lung metastases were further enhanced in 143B‐luc cells stably expressing the K112A mutant of Rab22a‐NeoF1 compared with those cells stably expressing Rab22a‐NeoF1 (Figure 2h,i, Supporting Information). The enhancement of metastasis was abolished by ectopic STUB1 in 143B‐Luc cells stably expressing Rab22a‐NeoF1 but not in those stably expressing the K112A mutant of Rab22a‐NeoF1 (Figure 2h,i, Supporting Information). Taken together, these results demonstrated that K112 is critical for STUB1‐mediated ubiquitination and degradation of Rab22a‐NeoF1 fusion protein.

**Figure 3:**
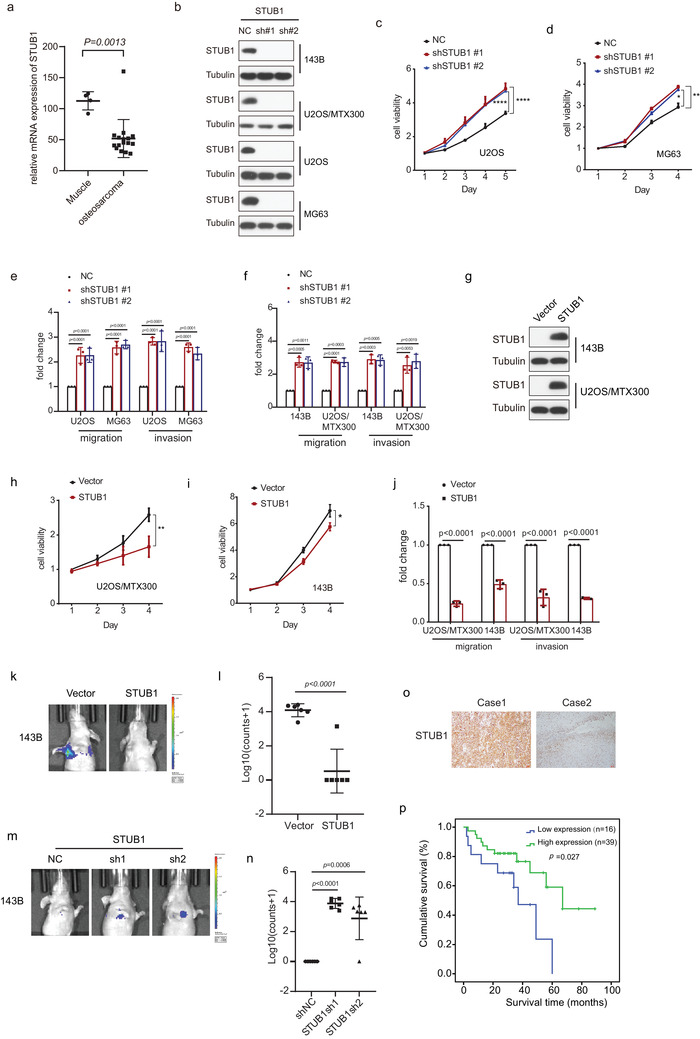
STUB1 acts as a tumor suppressor in osteosarcoma. a) The statistical results of STUB1 mRNA levels from our RNA‐seq data for 16 osteosarcoma tissues and 4 muscle tissues are shown. The data are presented as the mean ± SD. A two‐sided unpaired student's *t*‐test was performed, and *p* values are shown. b,g) The indicated stable cells were subjected to western blot analysis. The experiments were repeated three times independently with similar results. c,d,h,i) Cell viability was analyzed by MTT assay in the indicated stable cells. The depicted results are the averages of at least three independent experiments. The data are presented as the mean ± SD. Two‐way ANOVA test was performed, and *p* values are shown. *p < 0.05, **p < 0.01, ****p < 0.0001. e,f,j) Quantification analyses of migration and invasion assays using the indicated stable cell lines. The depicted results are the averages of at least three independent experiments. A two‐sided unpaired student's *t*‐test was performed, and *p* values are shown. k–n) The orthotropic osteosarcoma metastasis model in vivo using the indicated stable cell lines (*n* = 6 biologically independent animals). k,m) Representative images of mice. l,n) Quantification analyses of images. The data are presented as the mean ± SD. A two‐sided unpaired student's *t*‐test was performed, and *p* values are shown. o) Representative immunohistochemical staining images of STUB1 for 55 paraffin‐embedded osteosarcoma tissues. Scale bar: 100 µm. p) Overall survival curves were generated based on the protein levels of STUB1 in the osteosarcoma tissues using Kaplan–Meier plots. The Log‐rank test was performed, and *p* values are shown.

Because STUB1's function in osteosarcoma has not yet been determined, we sought to do so. As shown in **Figure** **3**a, the mRNA level of STUB1 was downregulated in osteosarcoma tissues compared with that in normal tissues from our RNA‐seq data.^[^ ^13^ ^]^ Knockout by sgRNA or knockdown by shRNA of STUB1 increased cell viability, migration, and invasion in U2OS, MG63, 143B, or U2OS/MTX300 cells (Figure 3b–f and Figure S3a,b, Supporting Information), whereas overexpression of STUB1 had the opposite effect in these cells (Figure 3g–j). Using the orthotopic osteosarcoma lung metastasis model with 143B‐luc cells in vivo, we determined that knockdown of STUB1 by shRNA increased and overexpression of STUB1 reduced lung metastasis, respectively (Figure 3k–n). Moreover, high protein level of STUB1 was associated with better overall survival in osteosarcoma patients (Figure 3o,p). Taken together, our results illustrate that STUB1 acts as a tumor suppressor in osteosarcoma.

### 2.3 NDP52 Acts as an Autophagy Receptor to Mediate the Lysosomal Degradation of Rab22a‐NeoF1 Fusion Protein

**Figure 4:**
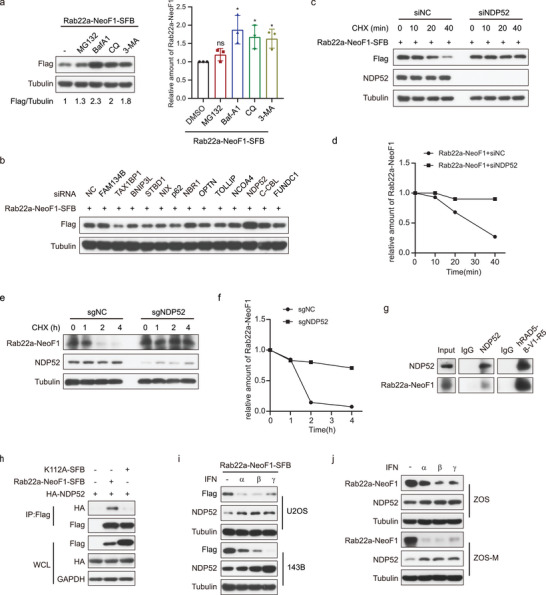
NDP52 as the autophagy receptor recognizes the ubiquitinated Rab22a‐NeoF1 fusion protein. a) U2OS cells stably expressing Rab22a‐NeoF1‐SFB were treated with MG132 (10 × 10^−6^ m), BafA1 (0.2 × 10^−6^ m), or 3‐MA (5 × 10^−3^ m) for 6 h or CQ (50 × 10^−6^ m) for 12 h, and then cell lysates were subjected to western blot. Left: western blot, right; quantification of western blotting. The experiments were repeated three times independently with similar results. The data are presented as the mean ± SD. A two‐sided unpaired student's *t*‐test was performed, and *p* values are shown. *p < 0.05, ns; no significance. b) 293T cells transfected with the indicted siRNAs for 24 h were transfected with the indicated plasmids for another 24 h. Cell lysates were subjected to western blotting. The experiments were repeated three times independently with similar results. c,d) 293T cells stably expressing Rab22a‐NeoF1‐SFB were transfected with the indicted siRNAs for 48 h, were treated with cycloheximide (CHX) for the indicated times, and then were analyzed by western blot. The experiments were repeated three times independently with similar results. d) Quantitation of the Rab22a‐NeoF1 protein levels in (c). e,f) ZOS cells with knockout of NDP52 by sgRNAs or sgNC as indicated were treated e) with cycloheximide (CHX: 20 µg mL^−1^) for the indicated time and subjected to western blot. f) Quantitation of the Rab22a‐NeoF1 protein levels in (e). The experiments were repeated three times independently with similar results. g) The co‐IPs were performed using ZOS‐M cells with IgG, hAb RAD5‐8, or anti‐NDP52 at their endogenous levels and were analyzed by western blot. The experiments were repeated three times independently with similar results. h) 293T cells co‐transfected with the indicated plasmids for 48 h were lysed and immunoprecipitated using anti‐FLAG beads followed by western blot analysis. The experiments were repeated three times independently with similar results. i,j) ZOS and ZOS‐M, as well as U2OS cells and 143B cells stably expressing Rab22a‐NeoF1‐SFB were treated with IFN*α*, *β*, and *γ* at 1000 u mL^−1^ for 18 h as indicated and then were subjected to western blot. The experiments were repeated three times independently with similar results.

Next, we looked into how STUB1 degrades Rab22a‐NeoF1 fusion protein in cells. As shown in **Figure** **4**a, the lysosome inhibitors, such as BafA1, CQ, and 3‐MA, could significantly increase the Rab22a‐NeoF1 protein level compared with the proteosome inhibitor MG132, indicating that the degradation of Rab22a‐NeoF1 predominantly occurs in lysosome. Then, each autophagy receptor, such as FAM134B, TAX1BP1, BNIP3L, STBD1, NIX, p62, NBR1, OPTN, TOLLIP, NCOA4, NDP52, C‐CBL, and FUNDC1, was knocked down by siRNA to examine which receptor may be involved in the degradation of Rab22a‐NeoF1 by lysosome. As shown in Figure 4b and Figure S4a in the Supporting Information, the Rab22a‐NeoF1 fusion protein was stabilized by knockdown of NDP52, but not other receptors, suggesting that NDP52 as the autophagy receptor may mediate the degradation of Rab22a‐NeoF1 by lysosome. Indeed, knockdown of NDP52 did stabilize exogenous and endogenous Rab22a‐NeoF1 fusion protein (Figure 4c–f). The interaction of Rab22a‐NeoF1 with NDP52 was detectable at their endogenous and exogenous levels (Figure 4g and Figure S4b,c, Supporting Information), and the CC domain, but not the SKICH or LIM‐L domain, of NDP52 was responsible for its binding to Rab22a‐NeoF1 (Figure S4d–f, Supporting Information). The K112A mutant of Rab22a‐NeoF1 that lacks the K63‐linked ubiquitin chains abrogated its binding to NDP52 (Figure 4h), which is consistent with the fact that autophagy receptor NDP52 generally recognizes cargos with the K63‐linked ubiquitin chains. In addition, because type I and II IFN have been reported to transcriptionally increase NDP52,^[^ ^14^ ^]^ IFN*α*, IFN*β*, and IFN*γ* could reduce ectopic and endogenous Rab22a‐NeoF1 protein levels (Figure 4i,j). These results revealed that the K63‐linked ubiquitination of Rab22a‐NeoF1 mediated by STUB1 is required for the NDP52‐dependent lysosomal degradation of Rab22a‐NeoF1 fusion protein.

### 2.4 PINK1 Phosphorylates Rab22a‐NeoF1 at Ser120 to Promote its Turnover by STUB1

**Figure 5:**
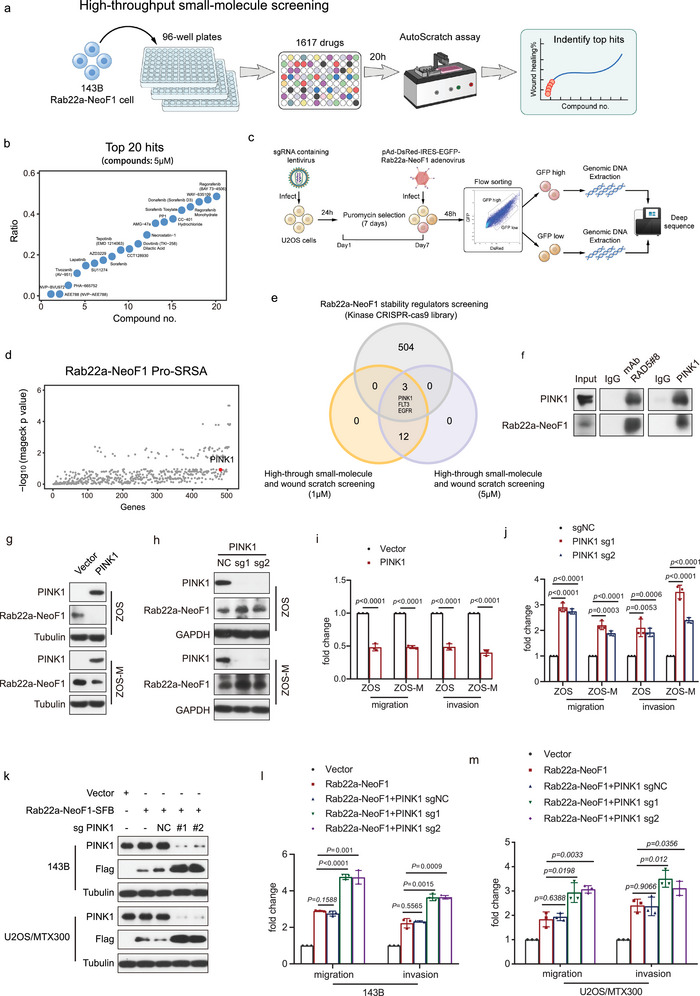
PINK1 acts as a tumor suppressor by regulating the stability of Rab22a‐NeoF1 fusion protein. a,b) Work flow of the high‐throughput kinase inhibitor drug (1617) screenings in 143B stably expressing Rab22a‐NeoF1‐SFB a) at two concentrations (1 × 10^−6^ and 5 × 10^−6^ m). Three replicates per drug. b) The top 19 hits are shown at the concentration of 5 × 10^−6^ m. c) Schematic of flow cytometry‐based Kinome CRISPR‐Cas9 knockout library screening pipeline and the gating strategy used in sorting cells. d) Scatterplot showing average log10 fold changes in mageck *p* value of sgRNA abundance in the GFP ^high^‐sorted population. sgRNAs targeting PINK1 are highlighted in red. Each dot represents an average of log10 fold changes in mageck *p* values for ten independent sgRNAs per gene. e) Venn diagram indicates the overlaps among the top 20 genes from the high‐throughput kinase inhibitor drug (1617) screenings and sgRNAs enriched from the Kinome CRISPR‐Cas9 knockout library screening. f) The co‐IPs were performed using ZOS‐M cells with IgG, mAb RAD5‐8, or anti‐PINK1 at their endogenous levels and were analyzed by western blot using hRAD5‐8‐V1‐R5 and PINK1. The experiments were repeated three times independently with similar results. g,h) The indicated stable ZOS and ZOS‐M cells with knockout or overexpression of PINK1 were analyzed by western blotting. The experiments were repeated three times independently with similar results. i,j,l,m) Quantification analyses of migration and invasion assays using the indicated stable cell lines. The depicted results are the averages of at least three independent experiments. The data are presented as the mean ± SD. A two‐sided unpaired student's *t*‐test was performed, and *p* values are shown. k) 143B and U2OS/MTX300 cells stably expressing vector or Rab22a‐NeoF1 were stably knocked out PINK1 with sgPINK1 or sgNC and then analyzed by western blot. The experiments were repeated three times independently with similar results.

Protein degradation is generally regulated by phosphorylation.^[^ ^15^ ^]^ To uncover the kinase(s) and their inhibitors that may be involved in the function and/or degradation of Rab22a‐NeoF1, we performed two screenings. One was the Rab22a‐NeoF1‐specific migration dependency Autoscratch assay in 143B cells stably expressing Rab22a‐NeoF1 to look for inhibitors that impair migration induced by Rab22a‐NeoF1 by using the high‐throughput small‐molecule screening of kinase inhibitor library (1617 compounds)^[^ ^16^ ^]^ at two concentrations (1 × 10^−6^ and 5 × 10^−6^ m) for 20 h (**Figure** **5**a). The top 20 hits drugs are shown after removing those that obviously inhibited proliferation, promoted apoptosis, or had high toxicity to cells in this kinase inhibitor screening (Figure 5b and Figure S5a and Table S5, Supporting Information). Another screening we used was the Pro‐SRSA with a CRISPR‐Cas9 kinase library using U2OS cells expressing DsRed‐IRES‐EGFP‐Rab22a‐NeoF1 to identify kinase(s) that regulate Rab22a‐NeoF1 protein stability (Figure 5c,d and Table S6, Supporting Information). By combining these two screenings, we got three kinase candidates, PINK1 (phosphatase and tensin honologue [PTEN]‐induced putative kinase 1), FLT3, and EGFR (Figure 5e). Knockdown of either PINK1 or EGFR, but not FLT3, stabilized Rab22a‐NeoF1 protein (Figure S5b,c, Supporting Information), whereas overexpression of PINK1, but not EGFR, decreased Rab22a‐NeoF1 protein (Figure S5d, Supporting Information).

Next, we investigated whether and how PINK1 regulates Rab22a‐NeoF1 fusion protein. The interaction of PINK1 with Rab22a‐NeoF1 fusion protein was detected easily at their ectopic and endogenous levels (Figure 5f and Figure S5e,f, Supporting Information). Knockout of PINK1 by sgRNA stabilized endogenous Rab22a‐NeoF1 fusion protein and promoted migration and invasion in both ZOS and ZOS‐M cells, whereas ectopic PINK1 had reverse effects in these cells (Figure 5g–j). Furthermore, knockout of PINK1 by sgRNA also stabilized ectopic Rab22a‐NeoF1 fusion protein, and further enhanced the migration and invasion in both 143B and U2OS/MTX300 cells stably expressing Rab22a‐NeoF1 (Figure 5k–m).

**Figure 6:**
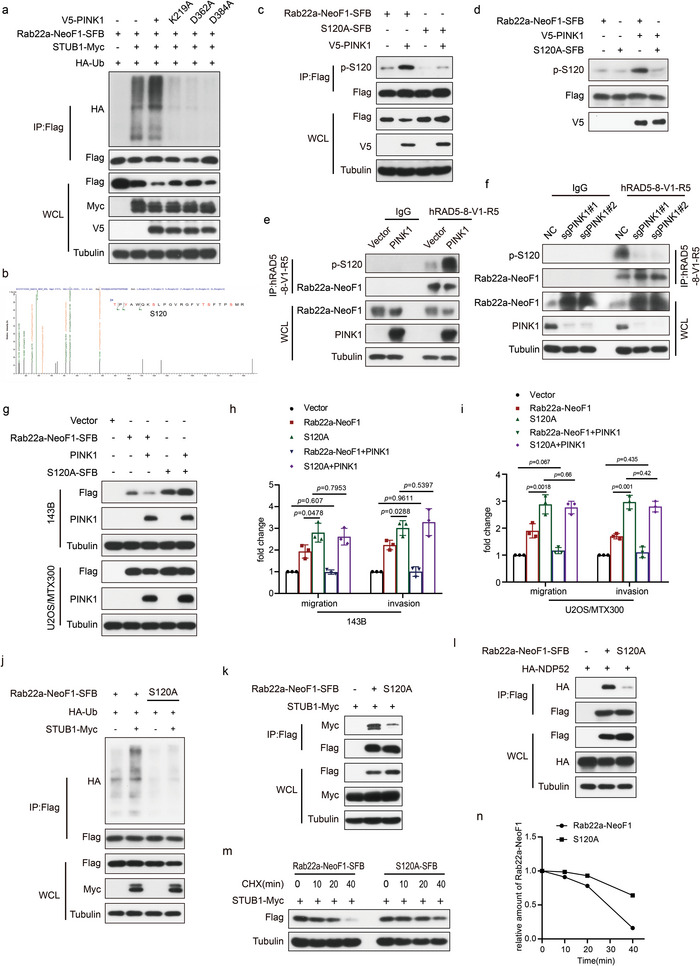
PINK1 phosphorylates Rab22a‐NeoF1 at Ser120 to promote its turnover by STUB1. a) 293T cells co‐transfected with the indicated plasmids for 48 h were analyzed by western blot. The experiments were repeated three times independently with similar results. b) The phosphorylation mass spectrometry of Rab22a‐NeoF1 protein as described in methods. c) 293T cells co‐transfected with the indicated plasmids for 48 h were lysed and immunoprecipitated using anti‐FLAG beads followed by western blot analysis. The experiments were repeated three times independently with similar results. d) Rab22a‐NeoF1‐SFB or its S120A mutant protein were purified from 293T cells and incubated with or without the purified V5 tagged PINK1 kinase in vitro as described in the methods and then analyzed by western blot. The experiments were repeated three times independently with similar results. e,f) Immunoprecipitation was performed using ZOS overexpression or knockout PINK1 cells with IgG and hAb RAD5‐8 then were analyzed by western blot. The experiments were repeated three times independently with similar results. g) The indicated stable cells were analyzed by western blot. The experiments were repeated three times independently with similar results. h,i) Quantification analyses of migration and invasion assays using the indicated stable cell lines in (g). The depicted results are the averages of at least three independent experiments. The data are presented as the mean ± SD. A two‐sided unpaired student's *t*‐test was performed, and *p* values are shown. j–l) 293T cells co‐transfected with the indicated plasmids for 48 h were lysed and immunoprecipitated using anti‐FLAG beads followed by western blot analysis. The experiments were repeated three times independently with similar results. m) 293T cells stably expressing Rab2a‐NeoF1 wild type or its K112A mutant as indicated were transfected with STUB1‐Myc for 48 h, treated with cycloheximide (CHX: 20 µg mL−1) for the indicated time, and then subjected to western blot. n) Quantitation of the Rab22a‐NeoF1 protein levels in (m). The experiments were repeated three times independently with similar results.

To explore how PINK1, a serine/threonine kinase, regulates Rab22a‐NeoF1 degradation, we used PINK1 mutants. As shown in **Figure** **6**a, the inactive kinase mutant forms of PINK1 including K219A, D362A, D384A diminished polyubiquitination of Rab22a‐NeoF1 mediated by STUB1, suggesting that PINK1 regulates Rab22a‐NeoF1 degradation dependent on its kinase activity. Next, mass spectrometry was performed to identify the phosphorylation site(s) of Rab22a‐NeoF1 by PINK1 (Figure 6b). Although five phosphorylation sites (Thr113, Ser120, Thr129, Thr130, and Ser134) were found, only the mutant S120A of Rab22a‐NeoF1 (Ser120 was mutated into alanine) was resistant to degradation mediated by PINK1 (Figure S6a, Supporting Information), indicating that PINK1 may phosphorylate Rab22a‐NeoF1 at Ser120. Indeed, using an antibody that specially recognizes the Ser120 phosphorylation of Rab22a‐NeoF1, the p‐S120 level of Rab22a‐NeoF1, but not of its S120A mutant, was enhanced by PINK1 in cells and in vitro kinase assay (Figure 6c,d). The p‐S120 level of endogenous Rab22a‐NeoF1 fusion protein was also increased and decreased by overexpression and knockout of PINK1 in ZOS cells, respectively (Figure 6e,f). Consistently, the protein level of Rab22a‐NeoF1, but not of its S120A mutant, was decreased by ectopic PINK1 (Figure 6g). Migration and invasion were further enhanced in both 143B and U2OS/MTX300 stably expressing the S120A mutant of Rab22a‐NeoF1 compared with those cells stably expressing Rab22a‐NeoF1. More importantly, the enhancement of both migration and invasion was abolished by ectopic PINK1 in both 143B and U2OS/MTX300 stably expressing Rab22a‐NeoF1 but not in those stably expressing the S120A mutant of Rab22a‐NeoF1 (Figure 6h,i).

Then, we tried to decipher how phosphorylation of Rab22a‐NeoF1 at Ser120 influences its stability. Compared with Rab22a‐NeoF1, the S120A mutant of Rab22a‐NeoF1 was much less polyubiquitinated and almost lost its binding to both STUB1 and NDP52 (Figure 6j–l), and consequently, the half‐life of this mutant protein was prolonged (Figure 6m,n).

Consistently, PINK1 might act as a tumor suppressor in osteosarcoma, as RNA‐Seq data^[^ ^13^ ^]^ showed that the mRNA level of PINK1 was decreased in osteosarcoma tissues compared with normal tissues and a high level of PINK1 predicted a good survival rate for osteosarcoma patients (Figure S6b,c, Supporting Information).

### 2.5 Both Sorafenib and Regorafenib Inhibit Osteosarcoma Lung Metastasis by Inducing PINK1 to Target the Lysosomal Degradation of Rab22a‐NeoF1 Fusion Protein

**Figure 7:**
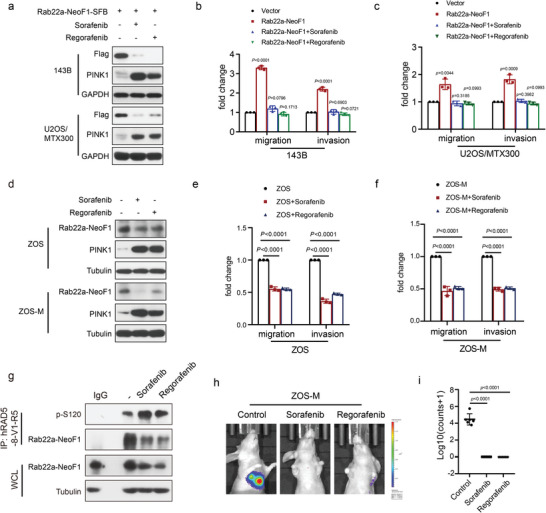
Sorafenib and Regorafenib inhibit lung metastasis of osteosarcoma by inducing PINK1 expression to degrade Rab22a‐NeoF1 fusion protein. a,d) 143B and U2OS/MTX300 cells stably expressing a) Rab22a‐NeoF1‐SFB, as well as d) ZOS and ZOS‐M cells were treated with Sorafenib (5 × 10^−6^ m) or Regorafenib (5 × 10^−6^ m) for 12 h as indicated, and cell lysates were subjected to western blotting. The experiments were repeated three times independently with similar results. b,c,e,f) Quantification analyses of migration and invasion assays using the indicated cells. The depicted results are the averages of at least three independent experiments. The data are presented as the mean ± SD. A two‐sided unpaired student's *t*‐test was performed, and *p* values are shown. g) ZOS cells were treated with Sorafenib (5 × 10^−6^ m) or Regorafenib (5 × 10^−6^ m) for 12 h then lysed and immunoprecipitated using anti‐hRAD5‐8‐V1‐R5 followed by western blot analysis. The experiments were repeated three times independently with similar results. h,i) The orthotropic osteosarcoma metastasis model in vivo using ZOS‐M cells under treatment of control, Sorafenib (5 × 10^−6^ m), or Regorafenib (5 × 10^−6^ m) (*n* = 6 biologically independent animals). h) Representative images of mice. i) Quantification analyses of lung metastases in mice.

Sorafenib and Regorafenib are two small molecule multi‐kinase inhibitors that have been reported to inhibit the progression of osteosarcoma.^[^ ^17^ ^]^ Sorafenib inhibits the activity of both electron transport chain and ATP synthase to activate the PINK1/Parkin pathway.^[^ ^18^ ^]^ Sorafenib and Regorafenib were ranked among the top 20 hits in our kinase inhibitor screening (Figure 5b and Figure S5a, Supporting Information). We therefore hypothesized that Sorafenib or Regorafenib would be used to target the lysosomal degradation of Rab22a‐NeoF1 to impede the functions of this fusion protein. As shown in Figure S7a–e in the Supporting Information, Sorafenib or Regorafenib induced PINK1 to decrease ectopic Rab22a‐NeoF1 fusion protein in a dose‐ and time‐dependent manner, and the autophagy inhibitors, such as Baf‐A1, CQ, and 3‐MA, could rescue the ectopic Rab22a‐NeoF1 protein levels in 143B cells treated by Sorafenib or Regorafenib. As shown in **Figure** **7**a–c, Sorafenib or Regorafenib induced PINK1 and decreased Rab22a‐NeoF1 fusion protein as well as abrogated the promotion of migration and invasion induced by Rab22a‐NeoF1 in both 143B and U2OS/MTX300 cells stably expressing Rab22a‐NeoF1. Likewise, Sorafenib or Regorafenib also induced PINK1 and increased p‐S120 of endogenous Rab22a‐NeoF1 fusion protein and consequently decreased endogenous Rab22a‐NeoF1 to diminish migration and invasion in ZOS or ZOS‐M cells (Figure 7d–g). Moreover, Sorafenib or Regorafenib was determined to decrease lung metastasis in mice using the orthotopic osteosarcoma lung metastasis model in vivo with ZOS‐M‐luc cells (Figure 7h,i).

In addition, both carbonyl cyanide m‐chlorophenyl hydrazone (CCCP) and valinomycin have also been reported to induce PINK1 activation.^[^ ^19^ ^]^ As shown in Figure S7f–k in the Supporting Information, CCCP or valinomycin induced PINK1 to degrade Rab22a‐NeoF1 fusion protein at exogenous and endogenous levels, which could be rescued by Baf‐A1 or CQ; the enhancement of migration and invasion was abrogated by CCCP or valinomycin in both 143B and U2OS/MTX300 stably expressing Rab22a‐NeoF1; CCCP or valinomycin also impaired migration and invasion in ZOS or ZOS‐M cells harboring endogenous Rab22a‐NeoF1.

**Figure 8:**
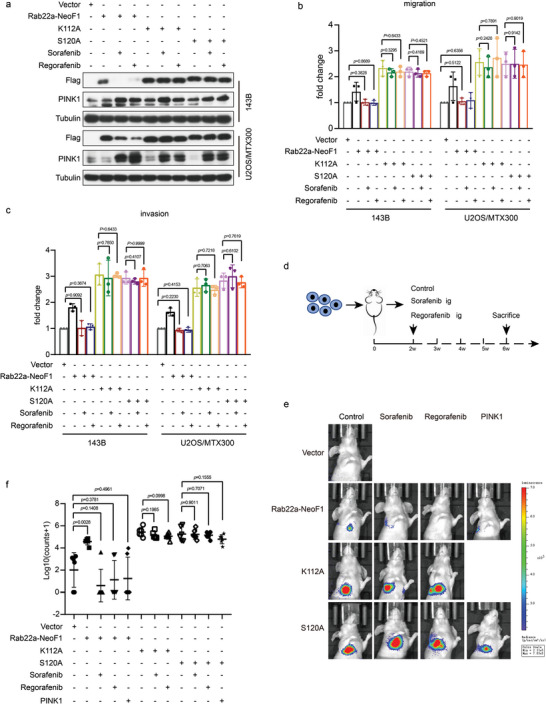
Sorafenib and Regorafenib inhibit osteosarcoma lung metastases through the PINK1/Rab22a‐NeoF1 axis. a) 143B and U2OS/MTX300 cells stably expressing Rab22a‐NeoF1‐SFB were treated with Sorafenib (5 × 10^−6^ m) or Regorafenib (5 × 10^−6^ m) for 12 h as indicated, and cell lysates were subjected to western blot. The experiments were repeated three times independently with similar results. b,c) Quantification analyses of migration and invasion assays using the indicated stable cells under treatment of control, Sorafenib (5 × 10^−6^ m), or Regorafenib (5 × 10^−6^ m). The depicted results are the averages of at least three independent experiments. The data are presented as the mean ± SD. A two‐sided unpaired student's *t*‐test was performed, and *p* values are shown. d) The procedure for in vivo orthotropic model of osteosarcoma metastasis under treatment of control, Sorafenib (5 × 10^−6^ m), or Regorafenib (5 × 10^−6^ m). e,f) In vivo orthotropic model of osteosarcoma metastasis using 143B‐Luc cells stably expressing Rab22a‐NeoF1 wild type (WT), K112A mutant, S120A mutant, WT plus PINK1, or S120A mutant plus PINK1 as indicated under treatment of control, Sorafenib (5 × 10^−6^ m), or Regorafenib (5 × 10^−6^ m) (*n* = 6 biologically independent animals). e) Representative images of mice. f) Quantification analyses of lung metastases in mice.

Furthermore, the enhancement of migration and invasion was abolished by Sorafenib or Regorafenib in both 143B and U2OS/MTX300 stably expressing Rab22a‐NeoF1 but not in those stably expressing S120A or K112A mutant of Rab22a‐NeoF1 (**Figure** **8**a–c). Using the orthotopic osteosarcoma lung metastasis model with 143B‐luc cells in vivo, lung metastases were determined to be further enhanced in 143B‐luc cells stably expressing either S120A or K112A mutant of Rab22a‐NeoF1 compared with those cells stably expressing Rab22a‐NeoF1. The enhancement of metastasis was abolished by Sorafenib or Regorafenib as well as ectopic PINK1 in 143B‐Luc cells stably expressing Rab22a‐NeoF1 but not in those stably expressing either S120A or K112A mutant of Rab22a‐NeoF1 (Figure 8d–f). Collectively, both Sorafenib and Regorafenib inhibited lung metastasis of osteosarcoma by inducing PINK1 to target the lysosomal degradation of Rab22a‐NeoF1 fusion protein.

## 3 Discussion

**Figure 9:**
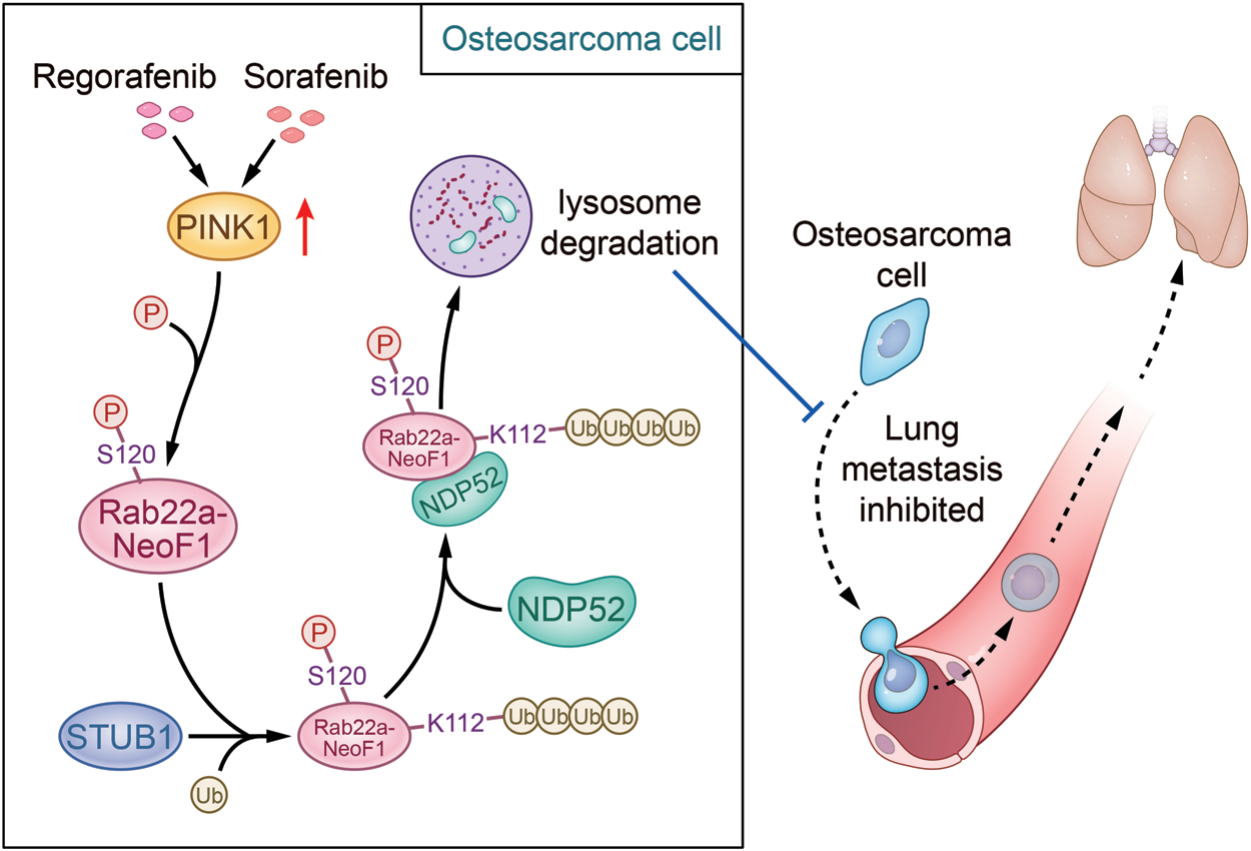
Proposed model for the lysosomal degradation of RAB22A‐NeoF1 fusion protein in osteosarcoma cells. In osteosarcoma cells, Rab22a‐NeoF1 fusion protein can be polyubiquitinated at lysine112 via the K63‐linked ubiquitin chains and then be recognized by autophagy receptor NDP52, leading to the lysosomal degradation of Rab22a‐NeoF1. This process is promoted by PINK1 kinase via phosphorylating Rab22a‐NeoF1 at serine120, and Sorafenib and Regorafenib can diminish osteosarcoma lung metastasis induced by Rab22a‐NeoF1 through upregulating PINK1.

In this report, as proposed in **Figure** **9**, we found that Rab22a‐NeoF1 fusion protein can be polyubiquitinated at lysine112 via the K63‐linked ubiquitin chains and then be recognized by autophagy receptor NDP52, leading to the lysosomal degradation of Rab22a‐NeoF1. This process is promoted by PINK1 kinase via phosphorylating Rab22a‐NeoF1 at serine120, and Sorafenib and Regorafenib can diminish osteosarcoma lung metastasis induced by Rab22a‐NeoF1 through upregulating PINK1. Our findings illustrate that the lysosomal degradation of Rab22a‐NeoF1 fusion protein is targetable for drug treatment of osteosarcoma lung metastasis, such as with Sorafenib and Regorafenib.

Fusion oncogenes are common in many cancer types, and many of them have been targeted with targeted drugs.^[^ ^20^ ^]^ For osteosarcoma metastasis, however, which is characterized by marked instability of its somatic genome, there are no efficient therapeutic targets. We have recently identified Rab22a‐NeoF1 fusion protein as promoting osteosarcoma lung metastasis,^[^ ^5^, ^6^ ^]^ and in this report we identified that Rab22a‐NeoF1 fusion protein was degraded by STUB1 E3 ligase, also known as carboxyl terminus of hsc70‐interacting protein (CHIP),^[^ ^21^ ^]^ which may function as a tumor suppressor in osteosarcoma. However, STUB1 (CHIP) has been considered a double‐edged sword in tumor development, depending on the cell types and substrates.^[^ ^22^ ^]^ We found that the K63‐linked ubiquitin chains of Rab22a‐NeoF1 fusion protein mediated by STUB1 are recognized by NDP52, an autophagy receptor, to be degraded by lysosome, and that IFN*α*, *β*, and *γ* increased NDP52 to reduce endogenous and exogenous Rab22a‐NeoF1 protein levels. These findings may be pathologically relevant, because type I and II IFN, which play crucial roles in osteosarcoma, can induce transcription of NDP52.^[^ ^14^ ^]^ In addition, STUB1 may be a target of covalent ISG15 conjugation, which is similar to ubiquitination and increases STUB1 E3 activity, and ISG15 is highly inducible by type I IFNs.^[^ ^23^ ^]^

PINK1 acts in a kinase‐dependent manner, mainly classified as having mitophagy‐dependent and ‐independent functions.^[^ ^24^ ^]^ PINK1 dysfunction is thought to be involved in cancers and other human diseases.^[^ ^25^ ^]^ For instance, PINK1 may be an oncogene or a tumor suppressor depending on the cancer type. PINK1 serves as a negative regulator of glioblastoma growth,^[^ ^26^ ^]^ but promotes proliferation and chemoresistance in lung cancer.^[^ ^27^ ^]^ In the present report, we have showed that PINK1 may be a tumor suppressor in osteosarcoma. More interestingly, we found that Rab22a‐NeoF1 fusion protein as a cytosolic protein is a new substrate for PINK1 kinase. This is consistent with an online paper showing that PINK1 kinase activity via phosphorylating a number of proteins in cytosol rather than its mitochondrial function is essential for the neuronal survival in primate brains,^[^ ^25c^ ^]^ although the classical roles of PINK1 are primarily related to its mitochondrial function.

More importantly, by two screenings of the kinase library and kinase inhibitors library, we discovered that Sorafenib and Regorafenib as multi‐kinase inhibitors diminished osteosarcoma lung metastasis induced by Rab22a‐NeoF1 fusion protein via the PINK1/Rab22a‐NeoF1 axis. Sorafenib inhibits the activity of both electron transport chain and ATP synthase to activate the PINK1, which is likely to be a general phenomenon.^[^ ^18^ ^]^ These findings are striking, as Sorafenib was able to temporarily inhibit osteosarcoma progression,^[^ ^17^, ^28^ ^]^ and Regorafenib was effective against recurrent and metastasis osteosarcoma.^[^ ^17b,c^ ^]^ We propose that Sorafenib and Regorafenib may benefit cancer patients who are positive for the *RAB22A*‐NeoF1 fusion gene.

## 4 Experimental Section

### Flow Cytometry‐Based PTM CRISPR‐Cas9 Screen

To produce virus, a CRISPR‐Cas9 knockout library, including sgRNAs targeting all E1, E2, and E3 ligases (709 genes); deubiquitinases (111 genes); (de)methytranferases (88 genes); and (de)acetyllationases (35 genes) was screened. Pooled plasmid was co‐transfected into HEK293T cells with lentiviral packaging plasmids psPAX2 and PMD2.G. HEK293T cells were cultured in D10 at 80% confluence at the time of transfection. Viral particles were harvested after 48 h. A total of 2 × 10^7^ U2OS cells were infected at MOI ∼ 0.3, and the guide representation was 500 cells per guide, selected by puromycin at 0.75 µg mL^−1^ for 7 days. These cells were reinfected with pAd‐DsRed‐IRES‐ EGFP‐Rab22a‐NeoF1 adenovirus at MOI ∼ 4.0 for 48 h to ensure that more than 95% of cells were positive. The MOI was measured as previously described.^[^ ^29^ ^]^ The cells were sorted into GFP high (5%) and GFP low (5%) populations using FACS. U2OS pAd‐DsRed‐IRES‐EGFP‐Rab22a‐NeoF1 cells with nontargeting sgRNA were used to guide gates for sorting.^[^ ^12^ ^]^

### sgRNA PCR Amplification and CRISPR Screen Data Analysis

Genomic DNA (gDNA) was isolated from GFP high and GFP low population cells using a TIANamp genomic DNA kit according to the manufacturer's protocol (Tiangen, Beijing, China). Two rounds of PCR were performed to generate the barcode‐indexed libraries for Illumina NovaSeq 6000 sequencing. The primers are listed in Table S3 in the Supporting Information. Raw sequence data were trimmed to 20 bp by removing a constant portion of the sgRNA sequences. MAGeCK (version 0.55) count command was used to map the trimmed sgRNA sequence to the lentiCRISPRv2 sgRNA library.

Raw sequence data were trimmed to 20 bp by removing a constant portion of the sgRNA sequences. MAGeCK (version 0.55) count command was used to map the trimmed sgRNA sequence to the mageck test sgRNA library. MAGeCK command was used to calculate the beta score for each gene with parameters “–norm‐method median –max‐sgrnapergene‐permutation 20.” A positive beta score means a gene is positively selected, and a negative beta score means a gene is negatively selected.

### pAd‐DsRed‐IRES‐EGFP‐Rab22a‐NeoF1 Adenovirus Production

Rab22a‐NeoF1 CDS was cloned into the pAd‐DsRed‐IRES‐EGFP adenoviral reporter vector,^[^ ^12^ ^]^ *Pac*Idigested and transiently transfected into HEK293A cells cultured in 6‐well plates for 2–3 weeks until a crude viral lysate could be harvested, which was amplified by infecting HEK293A cells.

### Cell Culture

Human cell lines U2OS, MG63, 143B, and HEK293T embryonic kidney cells were obtained from the American Type Culture Collection (ATCC) and cultured according to the instructions from the ATCC. ZOS and ZOS‐M cell line were two syngeneic human osteosarcoma cell lines from primary tumor and skip metastasis of one osteosarcoma patient, respectively, and were cultured as previously described.^[^ ^30^ ^]^ U2OS/MTX300 cell line was the MTX‐resistant U2OS cell line (MTX: 300 ng mL^−1^), and was cultured as previously described.^[^ ^30b^ ^]^ The 143B Luc, U2OS/MTX300 Luc, or ZOS‐M Luc cells were cultured as previously described,^[^ ^5^, ^31^ ^]^ and their derived stable cell lines were generated by infecting the lentiviruses carrying the related shRNAs, CRISPR guide RNAs, or cDNAs into cells that were selected with puromycin. All cell lines used in this study were authenticated using short‐tandem repeat profiling less than 6 months before this project was initiated, and they were not cultured for more than 1 month.

### Transfection Experiments

STUB1, FBXL12, PJA2, TRIM40, UBR2, ALG13, USP7, PINK1, FLT3, and EGFR siRNA library; FAM134B, TAX1BP1, NIX, TOLLIP, NCOA4, FUNDC1, p62, OPTN, NBR1, NDP52, C‐CBL, BNIP3L, and STBD1 autophagy receptor siRNA library was synthesized by RiboBio. Transfections were performed according to the manufacturer's instructions using Lipofectamine RNAiMAX transfection reagent (Invitrogen) and 50 nmol L^−1^ siRNA. Transient transfection of 293T cells was performed with polyethyleneimine (PEI, 25 kDa), and the cells were collected after 48 h for the following assay and for lentiviral packaging. The siRNAs used are listed in Table S2 in the Supporting Information.

### RNA Extraction and qRT‐PCR

Total RNA was prepared using an RNA extraction kit (TIANGEN), and cDNA was synthesized according to the manufacturer's instructions (Tarkara). qRT‐PCR was performed using a Light Cycler 480 instrument (Roche Diagnostics) with SYBR Green PCR Master Mix (Kapa). All reactions were carried out in a 10 µL reaction volume in triplicate. The primers for glyceraldehyde 3‐phosphate dehydrogenase (GAPDH) were obtained from Invitrogen. Standard curves were generated, and the relative amount of target gene mRNA was normalized to that of GAPDH. The specificity was verified by melting curve analysis. The primers used for qRT‐PCR are listed in Table S1 in the Supporting Information.

### Plasmids, Cloning, and Lentivirus Production

cDNAs for STUB1 or PJA2 were amplified by PCR and cloned into the pCDNA3.1 vector with the Myc tag. The HA tagged NDP52, V5 tagged PINK1 or EGFR were cloned into the PCDNA3.1 vector. Lentiviruses were produced by co‐transfection of plasmids psPAX2 and Pmd2.G into HEK293T cells as previously described.^[^ ^31^ ^]^ To generate STUB1 and PINK1 knockout human osteosarcoma cells with LentiCRISPR, guide RNA(gRNA) sequences were designed to target the coding sequence of STUB1 and PINK1 (http://crispr.mit.edu/). The shRNAs and sgRNAs are listed in Table S3 in the Supporting Information.

### Cell Viability and Proliferation Assays

A 3‐(4,5‐dimethylthiazol‐2‐yl)‐2,5‐diphenyltetrazolium bromide (MTT) assay was used to measure cell viability. Briefly, 143B and U2OS/MTX300 STUB1 overexpression cells, or U2OS and MG63 STUB1 knockdown cells were seeded at a density of 2000 cells per well in a 96‐well microplate. The cells were incubated with MTT for 4 h, and the optical density (OD) was detected at 490 nm with the microplate reader once per day for 4 days. The results were presented as the mean ± SD of three independent experiments.

### Boyden Chamber Assays

The migration and invasion were examined using 24‐well Boyden chambers with 8 µm inserts coated without (migration) or with Matrigel (invasion), as previously described.^[^ ^31^ ^]^ A total of 0.5 × 10^5^ (143B, U2OS, MG63) or 1.0 × 10^5^ (U2OS/MTX300, ZOS, ZOS‐M) per well were plated on the inserts and cultured at 37 °C in the upper chambers without serum for 10 or 18 h, respectively; the cells that crossed the inserts were stained with crystal violet (0.005%, Sigma) and counted under phase‐contrast microscopy.

### Ubiquitination Mass Spectrometry Analysis

Affinity purification of the cells overexpressing Rab22a‐NeoF1‐SFB and STUB1 was carried out. Briefly, 293T cells stably expressing Rab22a‐NeoF1‐SFB were transiently transfected with STUB1 for 36 h. The cells were lysed in NETN buffer containing 50 mmol L^−1^ *β*‐glycerophosphate, 10 mmol L^−1^ NaF, and 1 mg mL^−1^ each of pepstatin A and aprotinin. The lysates were centrifuged at 12 000 rpm to remove debris and then incubated with streptavidin‐conjugated beads (Amersham) for 4 h at 4 °C. The beads were washed five times with NETN buffer, followed by elution with NETN buffer containing 2 mg mL^−1^ biotin (Sigma). The elutes were incubated with S‐protein beads (Novagen) for 4 h. After five washes, the bound proteins were analyzed by sodium dodecyl sulfate‐polyacrylamide gel electrophoresis (SDS‐PAGE), and ubiquitination mass spectrometry was performed by APTBIO. The immunocomplexes were washed four times with NETN buffer and then subjected to SDS‐PAGE and western blotting.

### In Vitro Ubiquitination Assay

All reagents related to the in vitro ubiquitination assays were purchased from R&D Systems. For in vitro ubiquitination assays, Rab22a‐NeoF1‐SFB and STUB1‐Myc protein was affinity‐isolated from lysates of HEK293T cells using streptavidin‐conjugated beads (Amersham) or anti‐Myc affinity gel (CMCTAG) 48 h after transfected with the SFB‐tagged Rab22a‐NeoF1 and MYC‐tagged STUB1 plasmids, eluted by with biotin or Myc peptide overnight at 4 °C.

Then the affinity‐purified MYC‐tagged STUB1 protein was incubated with purified SFB‐tagged Rab22a‐NeoF1 protein in a 20 µL in vitro ubiquitination reaction mixture at 37 °C for 1 h. Then western blotting was subjected to detect the ubiquitination of the Rab22a‐NeoF1 protein. The in vitro ubiquitination reaction mixture contained 50 × 10^−3^ m Tris‐HCl, pH 7.5, 5 × 10^−3^ m MgCl_2_, 1 × 10^−6^ m ubiquitin, 20 × 10^−6^ m MG132, 2 × 10^−3^ m ATP, 2 × 10^−3^ m NaF, 1 × 10^−3^ m DTT (MP, 0210059780), 10 µg ubiquitin, 40 ng E1, and 200 ng E2. The ATP, ubiquitin, E1 enzyme, E2 enzyme and the reaction buffer were obtained from R&D.

### Phosphorylation Mass Spectrometry Analysis

Tandem affinity purification (TAP) was carried out as previously described.^[^ ^32^ ^]^ Briefly, HEK293T cells were infected with lentivirus containing SFB‐tagged Rab22a‐NeoF1 and V5‐tagged PINK1. The cells were lysed in NETN buffer containing 50 mmol L^−1^ *β*‐glycerophosphate, 10 mmol L^−1^ NaF, and 1 mg mL^−1^ each of pepstatin A and aprotinin. The lysates were centrifuged at 12 000 rpm to remove debris and then incubated with streptavidin‐conjugated beads (Amersham) for 4 h at 4 °C. The beads were washed five times with NETN buffer, followed by elution with NETN buffer containing 2 mg mL^−1^ biotin (Sigma). The elutes were incubated with S‐protein beads (Novagen) for 4 h. After five washes, the bound proteins were analyzed by SDS‐PAGE, and phosphorylation mass spectrometry was performed by APTBIO. The immunocomplexes were washed four times with NETN buffer and then subjected to SDS‐PAGE and western blotting.

### Generation of p‐S120 Antibody against S120 Phosphorylation of Rab22a‐NeoF1

p‐S120 antibody, an antibody specific for phosphorylation on Ser120 of Rab22a‐NeoF1, was generated by immunizing rabbits with the coupled peptide CYAWQK‐(phospho)S‐LPGVR.

### Immunoblot and Immunoprecipitation

For western blot analysis, the cells were lysed in radioimmunoprecipitation assay buffer (RIPA) buffer (50 × 10^−3^ m Tris‐HCl, 150 × 10^−3^ m NaCl, 5 × 10^−3^ m ethylenediaminetetraacetic acid (EDTA), 0.5% NP‐40) containing protease inhibitor and phosphatase inhibitor cocktails (Thermo scientific). Lysates were cleared by centrifugation at 12 000 rpm for 20 min at 4 °C. The lysates were first incubated with antibody beads or agarose overnight at 4 °C, or antibodies overnight at 4 °C followed by incubation with protein A/G PLUS‐Agarose (Santa Cruz) at 4 °C for 2 h. Then the precipitates were washed five times with cold RIPA buffer and eluted with 5 x SDS sample buffer. The immunoprecipitates were separated by SDS‐PAGE and transferred to a polyvinylidene fluoride membrane (Millipore). The membranes were blocked in Tris‐buffered saline (TBS) with 5% nonfat milk and 0.1% Tween 20, probed with primary antibodies overnight at 4 °C, washed five times by TBS with 5% nonfat milk and 0.1% Tween 20, and then incubated with secondary horseradish‐peroxidase‐conjugated antibodies (Promega). ClarityTM Western ECL substrate (Bio‐Rad) was then used for detection.

### High‐Throughput Compound Screening

High‐throughput screening of the Kinase Inhibitor Library (96‐well) (Selleck, L1200), which includes 1617 kinase inhibitor compounds, was carried out using wound scratch assay in a 96‐well format. Briefly, 143B Rab22a‐NeoF1 expression cells were cultured in 80–90% confluence in a 96‐well plate (*n* = 3), and Autoscratch assay was performed the next day. Then, the Kinase inhibitors with two concentrations (1 × 10^−6^ and 5 × 10^−6^ m) or dimethyl sulfoxide (DMSO) control were added into each well, respectively. The plate was incubated and monitored with the high content screening system ImageXpress MicroConfocal for 24 h (molecular devices). The wound scratch width was calculated by MetaXpress software.

### Kinase Assay In Vitro

293T cells overexpressing SFB‐tagged Rab22a‐NeoF1 or V5‐tagged PINK1 were lysed in cell lysis buffer (20 × 10^−3^ m Tris [pH 7.5], 150 × 10^−3^ m NaCl, 1 × 10^−3^ m EDTA, 1 × 10^−3^ m EGTA, 1% Triton X‐100, 2.5 × 10^−3^ m sodium pyrophosphate, 1 × 10^−3^ m *β*‐glycerophosphate, 1 × 10^−3^ m Na_3_VO_4_, and 1 µg mL^−1^ Leupeptin) containing protease inhibitor and phosphatase inhibitor cocktails (Thermo scientific). The lysates were first incubated with streptavidin‐conjugated beads (Amersham) or V5‐conjugated beads (Thermo scientific) overnight at 4 °C. The beads were washed five times with NETN buffer, followed by elution with NETN buffer containing 2 mg mL^−1^ biotin (Sigma) or V5 peptide. The elutes containing Rab22a‐NeoF1‐SFB and V5‐tagged PINK1 were added into the kinase reaction system with 200 mµ ATP to incubate for 30 min at 30 °C and then subjected to SDS‐PAGE and western blotting.

### Xenograft Experiments

All animal experiments were approved by the Animal Research Committee of Sun Yat‐sen University Cancer Center (No# L102012020001Z) and performed in accordance with established guidelines. For the in vivo orthotopic osteosarcoma metastasis model, as previously described,^[^ ^5^ ^]^ male 4–6 weeks old BALB/c nude mice (6 mice per group) were used for 143B luc and ZOS‐M luc cells. Mice were implanted with cells infected with the indicated constructs. Drug administration began 2 weeks post‐injection when the tumors were deemed palpable. The Sorafenib was dissolved in a vehicle containing 45% PEG400 and 5% DMSO and 50% H_2_O and was administrated via intragastric administration (ig) at 30 mg kg^−1^ every day to 6 weeks. Regorafenib was dissolved in a vehicle containing 30% PEG300 and 2% DMSO and 5% Tween 80 and 63% H_2_O and was administrated via intragastric administration (ig) at 10 mg kg^−1^ every day to 6 weeks. The mice were monitored for lung metastases using the IVIS Lumina Imaging System, and their body weights were monitored weekly. The animals were sacrificed when their tumor size reached 1.5 cm in diameter, and the lungs were harvested for hematoxylin and eosin staining.

### Human Tissue Specimens

The Institutional Review Board of Sun Yat‐sen University Cancer Center approved this study (No# GZR2020‐284). A total of 55 paraffin‐embedded primary specimens were obtained from the recruited osteosarcoma patients. The patients were diagnosed according to their clinicopathological characteristics at the First Affiliated Hospital of Sun Yat‐sen University from 2007 to 2014. No patients had received radiotherapy and/or chemotherapy prior to surgery. Tumors were staged according to the Union for International Cancer Control TNM staging system. Resected specimens were macroscopically examined to determine the location and size of a tumor, and specimens for histology were fixed in 10% vol/vol formalin and processed for paraffin embedding.

### Immunohistochemistry Staining (IHC)

IHC staining was performed using standard procedures. Slides were blocked using a blocking solution and incubated overnight with primary antibodies. The primary antibodies against STUB1 (Novus Biotech) were diluted 1:100. The tissue slides were treated with a nonbiotin horseradish peroxidase detection system according to manufacturer's instructions (Dako). IHC staining was evaluated by two independent pathologists. The protein expression levels of STUB1 were evaluated based on 13 scores. To evaluate STUB1, a semiquantitative scoring criterion was used in which both the staining intensity and positive areas were recorded. A staining index (values 0–12), which was obtained as the product of the intensity of positive staining (weak, 1; moderate, 2; strong, 3) and the proportion of immunopositive cells of interest (0%, 0; <10%, 1; 10–50%, 2; 51–80%, 3; >80%, 4), was calculated. The immunohistochemical cut‐off for high or low expression of the indicated molecule was determined based on the receiver operating characteristic (ROC) curve analysis. The sensitivity and specificity for discriminating dead or alive was plotted as the IHC score, thus generating a ROC curve. The cut‐off value was established as the point on the ROC curve where the sum of sensitivity and specificity was maximized. Cancers with scores above the obtained cut‐off value were considered to have high expression of the indicated molecule and vice versa.

### Statistical Analysis

SPSS software (version 16.0, SPSS Inc., Chicago, IL, USA) and GraphPad Prism 8 (GraphPad software, San Diego, CA, USA) were used for the statistical analysis. The mean values obtained for the control and experimental groups were analyzed for significant differences. The data were presented as the mean ± SD. The error bars indicated the SD. Unpaired Student's *t*‐test (two‐tailed) and two‐way analysis of variance (ANOVA) were used to compare the statistical significance of differences between groups. STUB1 expression and overall survival curves were assessed by Kaplan–Meier plots and compared using a log‐rank test. Differences were considered significant when *p* values were < 0.05, and *p < 0.05, **p < 0.01, ***p < 0.001, and ****p < 0.0001.

## Conflict of Interest

The authors declare no conflict of interest.

## Author Contributions

C.Z., L.Z., and W.L. contributed equally to this work. T.K. and D.L. conceived the idea and wrote the manuscript. D.L. and L.Z. designed the experiments, analyzed the data, interpreted the results, and performed the most experiments. W.L. performed the flow and interpreted the results. Y.Z. and C.Z. cloned the plasmids and purified the protein. X.W. and R.Z. collected clinical samples and performed the immunohistochemical data. All co‐authors had seen and approved the manuscript.

## Supporting information

Supporting Information

Supplemental Table 1

Supplemental Table 2

Supplemental Table 3

Supplemental Table 4

Supplemental Table 5

Supplemental Table 6

## Data Availability

The data that support the findings of this study are available in the supplementary material of this article.
