# Supplementary material for: Targeting the Lysosomal Degradation of Rab22a‐NeoF1 Fusion Protein for Osteosarcoma Lung Metastasis
Source: Adv Sci (Weinh). 2022 Dec 18;10(5):2205483. doi: 10.1002/advs.202205483 (PMC9929137; doi:10.1002/advs.202205483)
Supplement: Supplementary file 1 — Supporting Information [file ADVS-10-2205483-s004.pdf]

ADVANCED SCIENCE
Open Access

Supporting Information

for *Adv. Sci.*, DOI 10.1002/advs.202205483

Targeting the Lysosomal Degradation of Rab22a-NeoF1 Fusion Protein for Osteosarcoma Lung Metastasis

Cuiling Zeng, Li Zhong, Wenqiang Liu, Yu Zhang, Xinhao Yu, Xin Wang, Ruhua Zhang, Tiebang Kang and Dan Liao**

Supplemental Figures

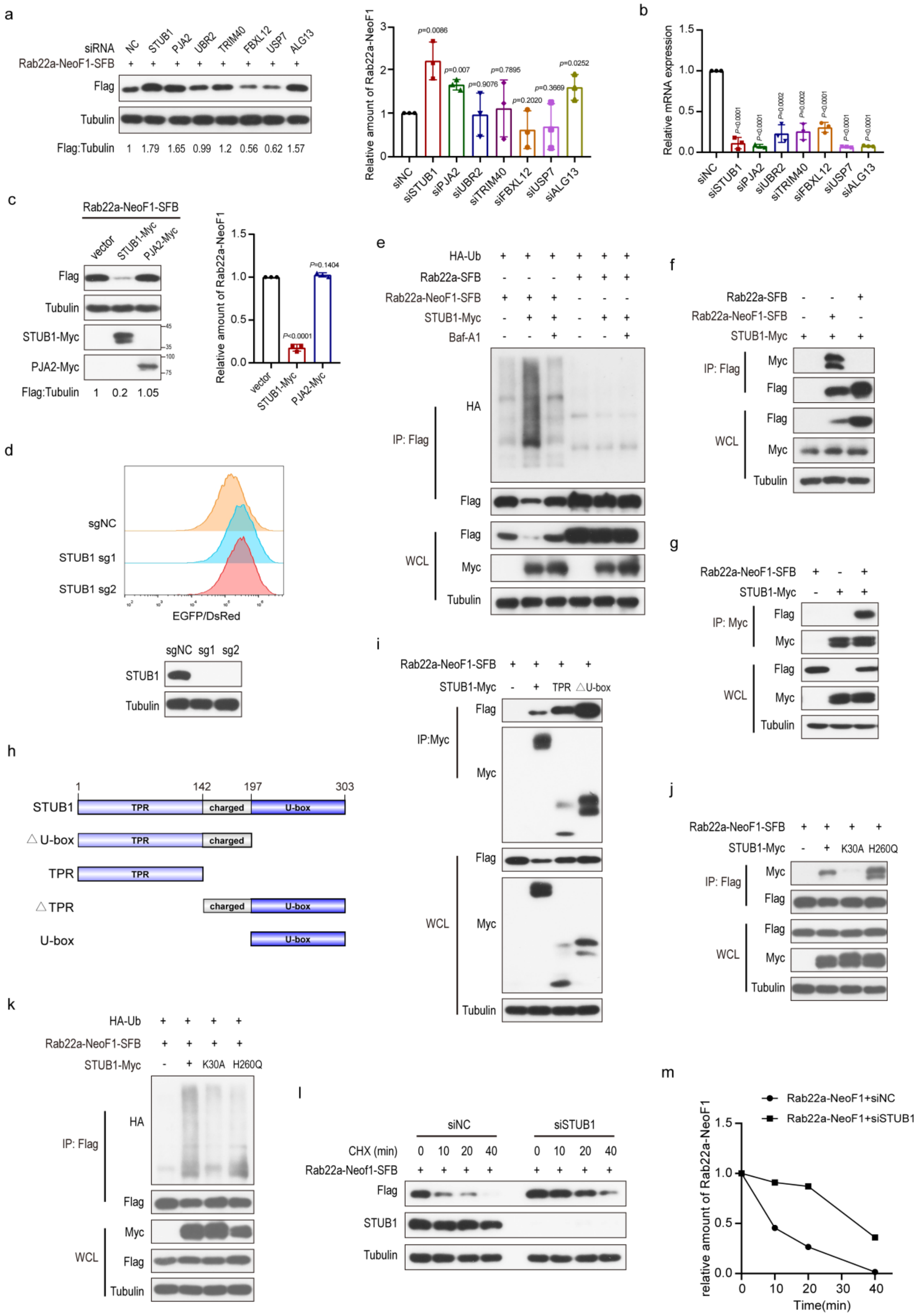

Supplementary Figure 1. STUB1 acts as an E3 ligase targeting Rab22a-NeoF1 fusion protein in osteosarcoma.

a-b, U2OS cells stably expressing Rab22a-NeoF1-SFB were transfected with siRNAs targeting the indicated genes for 48 h and then subjected to western blot (**a**, left; western blot, right; quantifition of western blotting) and qPCR (**b**). NC: negative control. The experiments were repeated three times independently with similar results. The data are presented as the mean ± SD. A two-sided unpaired student's t-test was performed, and *p* values are shown.

c, e–g, i–k, 293T cells co-transfected with the indicated plasmids for 48 h, treated with or without Baf-A1(200nM) for 6 h (**e**), were lysed and subjected to western blot (**c**, left; western blot, right; quantifition of western blotting) or were immunoprecipitated using anti-FLAG or anti-Myc beads followed by western blot analysis as indicated (**e–g, i–k**). The experiments were repeated three times independently with similar results. The data are presented as the mean ± SD. A two-sided unpaired student's t-test was performed, and *p* values are shown.

d, U2OS cells stably infected with pAd-DesRes-IRES-EGFP-Rab22a-NeoF1 were knocked out STUB1 with sgSTUB1 or sgNC and then were then analyzed by flow cytometry (top panel) or subjected to western blot (bottom panel). The experiments were repeated three times independently with similar results.

h, The fragments of STUB1 plasmids.

l, m, U2OS cells stably expressing Rab22a-NeoF1-SFB were knocked down STUB1 by siRNAs or siNC (**k, l**) for 48 h as indicated. They were then treated with cycloheximide (CHX: 20 μg/ml) for the indicated time and subjected to western blot (**k**). Quantitation of the Rab22a-NeoF1 protein levels in **k** (**l**). The experiments were repeated three times independently with similar results.

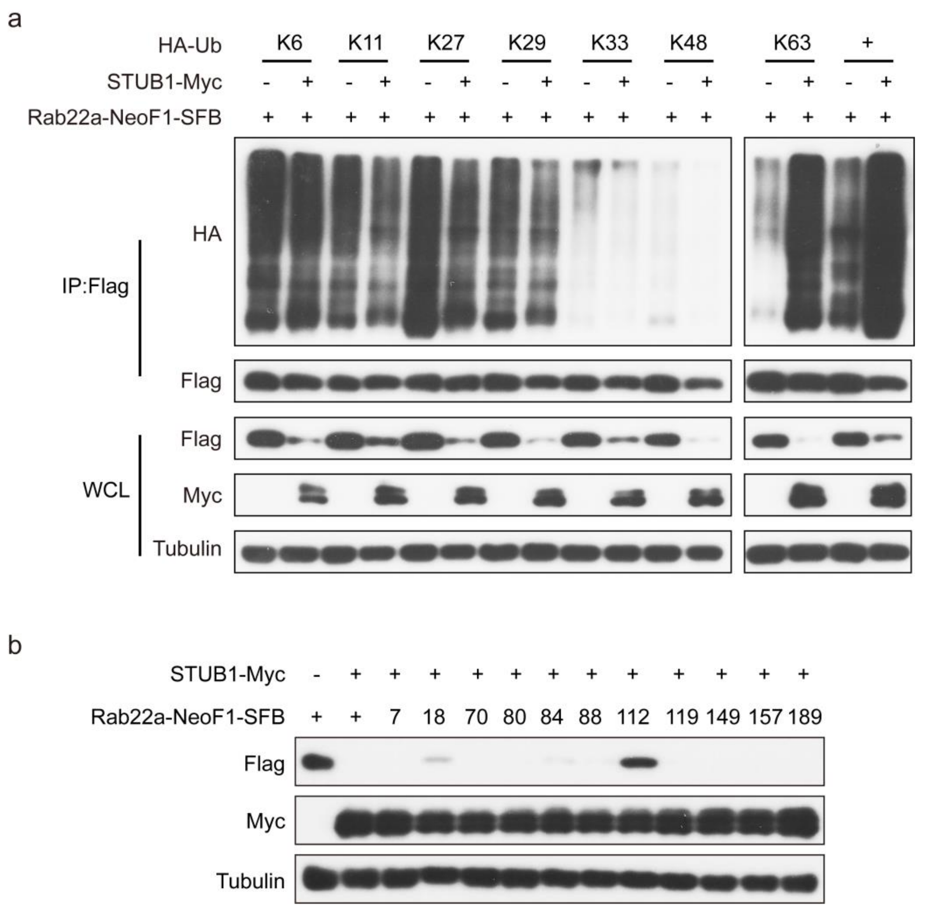

Supplementary Figure 2. STUB1 polyubiquitinates Rab22a-NeoF1 at K112.

a, 293T cells co-transfected with the indicated plasmids for 48 h were lysed and were immunoprecipitated using anti-FLAG or anti-Myc beads followed by western blot analysis. Data representative of three independent experiments.

b, 293T cells co-transfected STUB1-Myc with Rab22a-NeoF1-SFB or its alanine mutants at the indicated sites of lysine residue for 48 h were analyzed by western blot. Data are representative of three independent experiments.

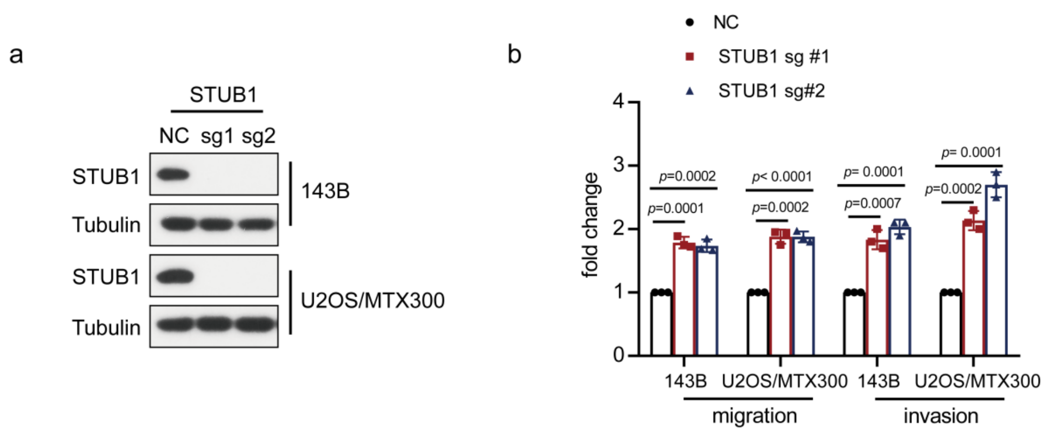

Supplementary Fig. 3. STUB1 may be a tumor suppressor in osteosarcoma.

a, 143B and U2OS/MTX300 cells stably knocked out of STUB1 with sgRNAs were subjected to western blot analysis. NC: negative control. Data are representative of three independent experiments.

b, Quantification analyses of migration and invasion assays using the indicated stable cells in **a**. The data are presented as the mean ± SD. A two-sided unpaired student's t-test was performed, and *p* values are shown.

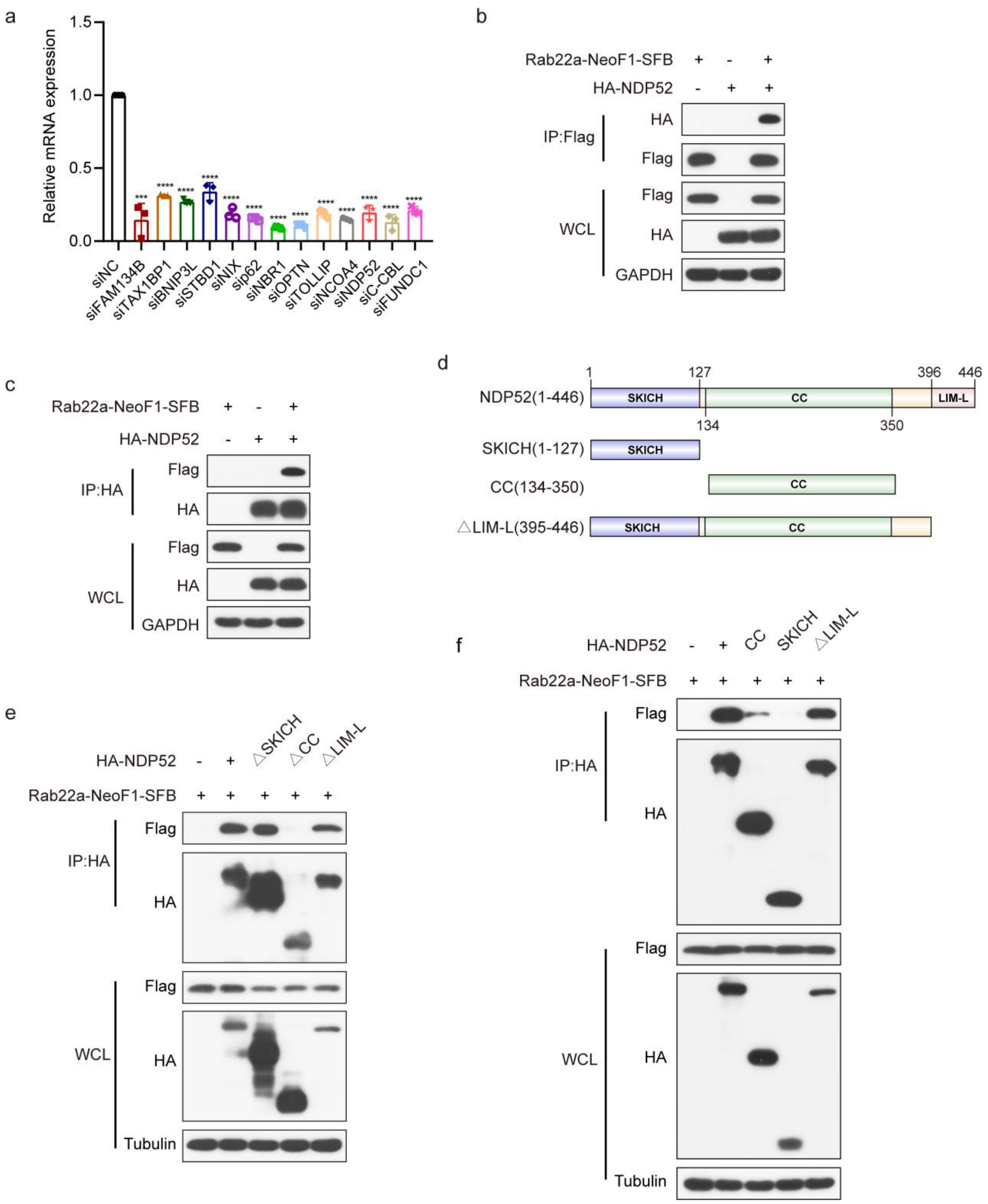

Supplementary Fig. 4. Rab22a-NeoF1 fusion protein interacts with the CC domain of NDP52.

a, Quantification analysis of knockdown efficiencies by real-time PCR of autophagy receptor mRNA levels as indicated in 293T cells. The depicted results are the averages of three independent experiments. The data are presented as the mean ± SD. A two-sided unpaired student's t-test was

performed, and *p* values are shown. ***$p<0.001$, ****$p<0.0001$.

b, c, e, **f**, 293T cells co-transfected with the indicated plasmids for 48 h were lysed and immunoprecipitated using anti-FLAG or anti-HA beads, followed by western blot as indicated. The experiments were repeated three times independently with similar results.

d, The fragments of NDP52 plasmids.

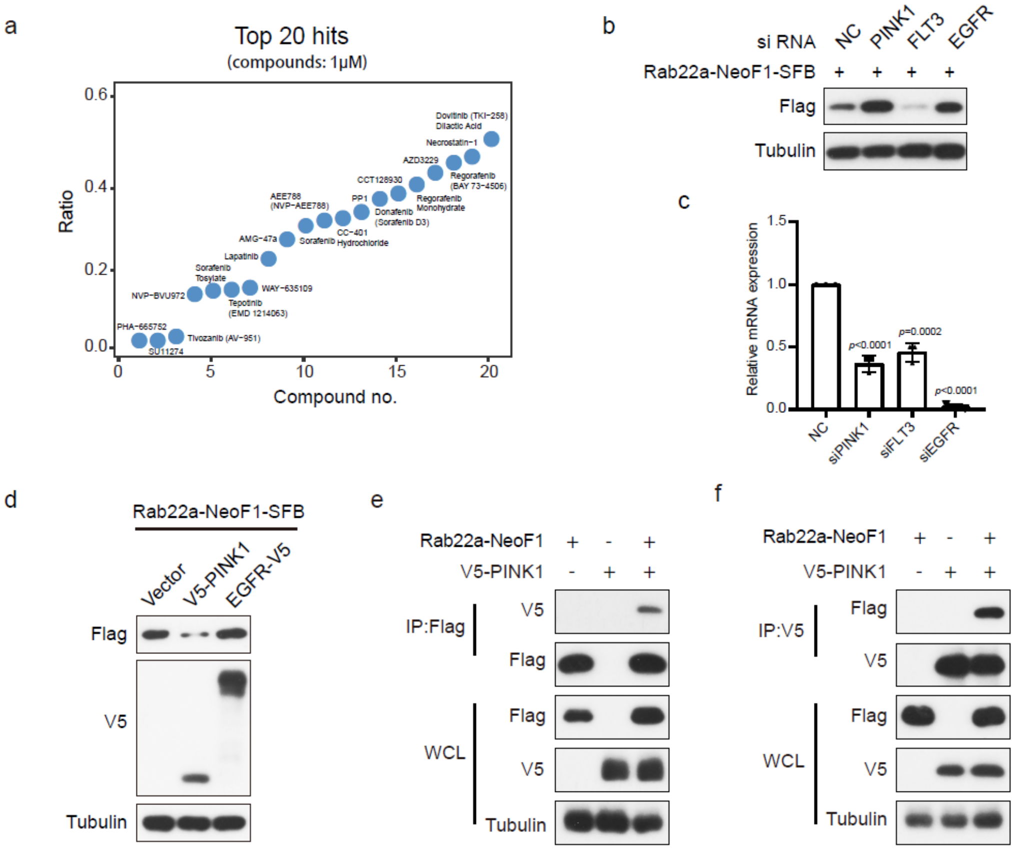

Supplementary Fig. 5. PINK1 kinase interacts with and down-regulates Rab22a-NeoF1 fusion protein.

a, The top 20 hits are shown from the High-throughput kinase inhibitor drug (1,617) screenings at the concentration of 1 μM in 143B stably expressing Rab22a-NeoF1-SFB. Three replicates per drug were used.

b, c, U2OS cells stably expressing Rab22a-NeoF1-SFB were transfected with siRNAs targeting PINK1, FLT3, EGFR, or negative control (NC) for 48 h and then analyzed by western blot (top panel) and qPCR (bottom panel). The experiments were repeated three times independently with similar results. The data are presented as the mean ± SD. A two-sided unpaired student's t-test was performed, and *p* values are shown.

d-f, 293T cells co-transfected with the indicated plasmids for 48 h were lysed

and subjected to western blot (**d**) or were immunoprecipitated using anti-FLAG or anti-V5 beads followed by western blot as indicated (**e, f**). The experiments were repeated three times independently with similar results.

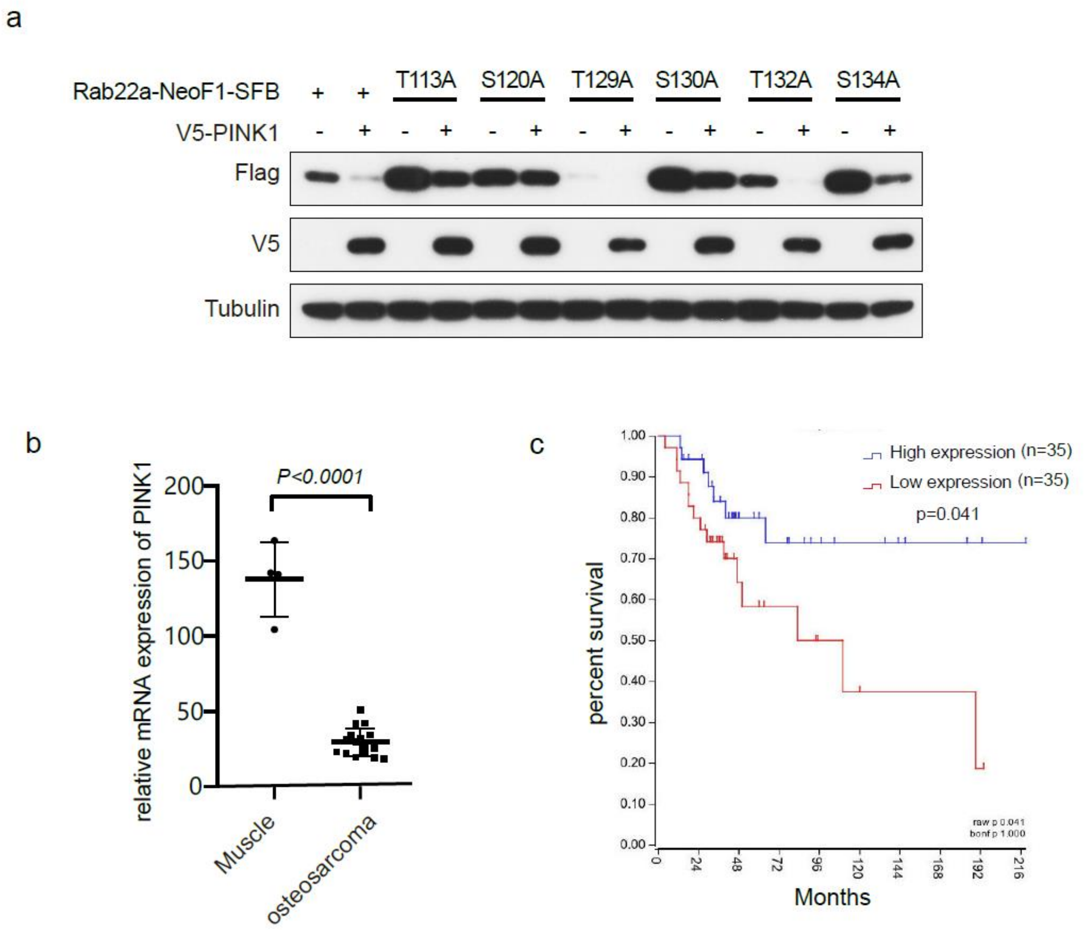

Supplementary Fig. 6. PINK1 can phosphorylate Rab22a-NeoF1 at Serine 120 to promote its degradation and may be a tumor suppressor in osteosarcoma.

a, 293T cells co-transfected with the indicated plasmids for 48 h were subjected to western blot. The experiments were repeated three times independently with similar results.

b, The statistical results of PINK1 mRNA levels from our RNA-Seq data for 16 osteosarcoma tissues and 4 normal tissues are shown.

c, Overall survival curves of osteosarcoma patients were generated based on the protein levels of PINK1 in R2 database using Kaplan-Meier plots. The Log-rank test was performed, and *p* values are shown.

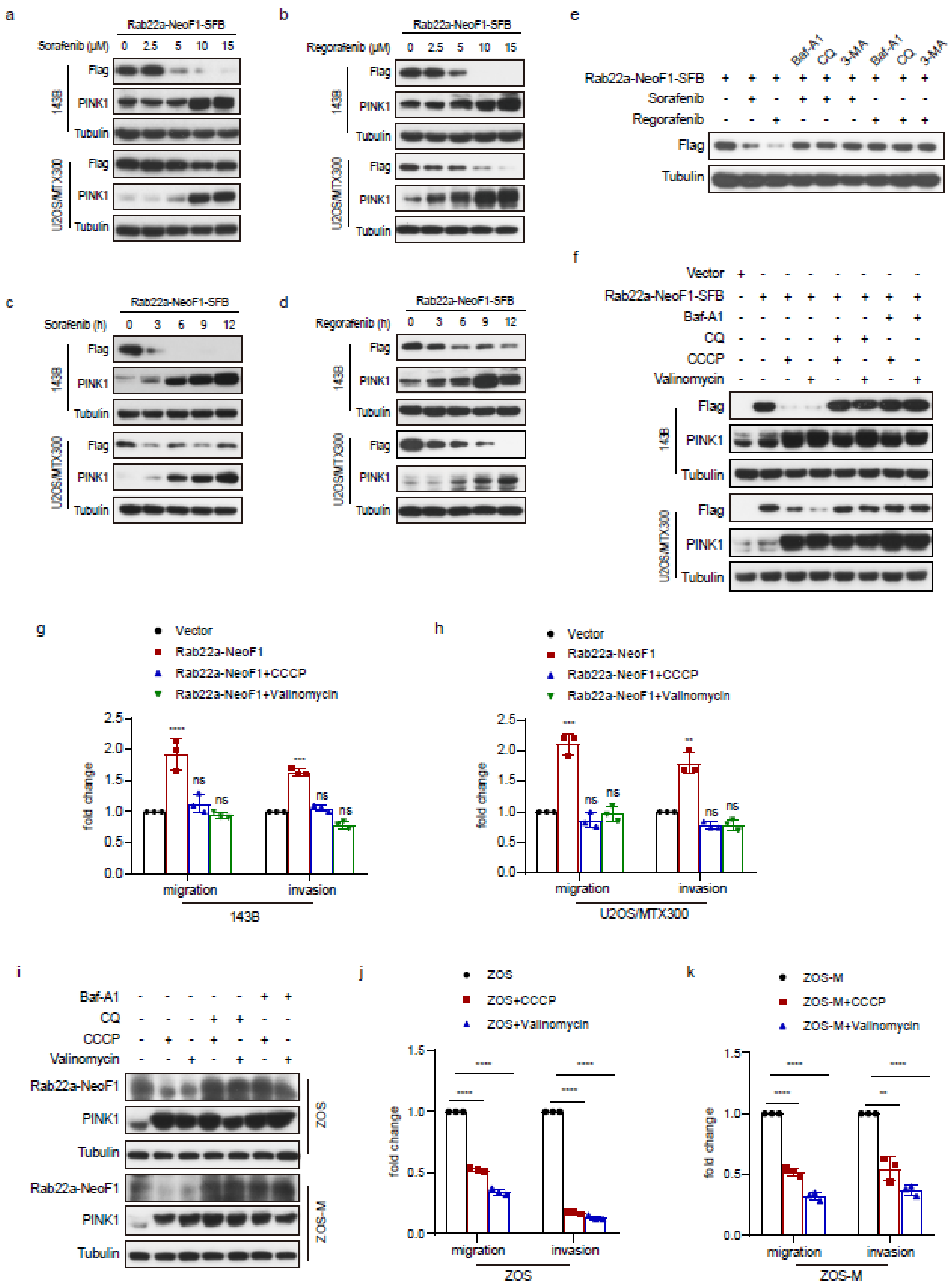

Supplementary Fig. 7. Sorafenib and Regorafenib induce PINK1 to decrease ectopic Rab22a-NeoF1 fusion protein in a dose- and time-dependent manner.

a-d, 143B cells and U2OS/MTX300 cells stably expressing Rab22a-NeoF1-SFB were treated with Sorafenib or Regorafenib at the indicated concentrations for 12 h (**a, b**) or at the concentration of 5μM for the indicated times (**c, d**) and were then analyzed by western blot. The experiments were repeated three times independently with similar results.

e, 143B cells stably expressing Rab22a-NeoF1-SFB were treated with Sorafenib or Regorafenib at the indicated concentrations for 12 h, with or without Baf-A1 (0.2μM, 6h), CQ (50μM, 12h), 3-MA (5mM, 6h), and were then analyzed by western blot. The experiments were repeated three times independently with similar results.

f,i, 143B and U2OS/MTX300 cells stably expressing Rab22a-NeoF1-SFB (**f**), as well as ZOS and ZOS-M cells (**i**) were treated with CCCP or valinomycin at the concentrations of 5μM for 6 h, with or without Baf-A1 (0.2μM, 6h), CQ (50μM, 12h) as indicated, and cell lysates were subjected to western blotting. The experiments were repeated three times independently with similar results.

g,h,j,k, Quantification analyses of migration and invasion assays using the indicated stable cell lines. The depicted results are the averages of at least three independent experiments. The data are presented as the mean ± SD. A two-sided unpaired student's t-test was performed, and *p* values are shown. ********$p<0.0001$, *******$p<0.001$, ******$p<0.01$, ns, no significance.
