# Supplementary material for: Targeting the Lysosomal Degradation of Rab22a‐NeoF1 Fusion Protein for Osteosarcoma Lung Metastasis
Source: Adv Sci (Weinh). 2022 Dec 18;10(5):2205483. doi: 10.1002/advs.202205483 (PMC9929137; doi:10.1002/advs.202205483)
Supplement: Supplementary file 2 — Supplemental Table 1 [file ADVS-10-2205483-s003.pdf]

ADVANCED SCIENCE
Open Access

Supporting Information

for *Adv. Sci.*, DOI 10.1002/advs.202205483

Targeting the Lysosomal Degradation of Rab22a-NeoF1 Fusion Protein for Osteosarcoma Lung Metastasis

Cuiling Zeng, Li Zhong, Wenqiang Liu, Yu Zhang, Xinhao Yu, Xin Wang, Ruhua Zhang, Tiebang Kang and Dan Liao**

Table 1

Oligonucleotides	Target
p62-F	GACTACGACTTGTGTAGCGTC
p62-R	AGTGTCCGTGTTTCACCTTCC
STBD1-F	GACCTTGGTAAACTGCAAGCA
STBD1-R	CCTTTTTGGAATCCCCATTCTCC
NDP52-F	TGAAGGAGGCGCAAGACAAAA
NDP52-R	CATCTGCTGTTGCTCCAAGGT
C-CBL-F	TAGGCGAAACCTAACCAAACTG
C-CBL-R	AGAGTCCACTTGGAAAGATTCCT
BNIP3L-F	TTGGATGCACAACATGAATCAGG
BNIP3L-R	TCTTCTGACTGAGAGCTATGGTC
OPTN-F	CCAAACCTGGACACGTTTACC
OPTN-R	CCTCAAATCTCCCTTTCATGGC
NBR1-F	CCAAACCTGGACACGTTTACC
NBR1-R	CCTCAAATCTCCCTTTCATGGC
GAPDH-F	ATCACCATCTTCCAGGAGCGA
GAPDH-R	CCTTCTCCATGGTGGTGAAGAC
PINK1-F	GCCTCATCGAGGAAAAACAGG
PINK1-R	GTCTCGTGTCCAACGGGTC
FLT3-F	CTGAATTGCCAGCCACATTTTG
FLT3-R	GGAACGCTCTCAGATATGCAG
EGFR-F	AGGCACGAGTAACAAGCTCAC
EGFR-R	ATGAGGACATAACCAGCCACC
UBR2-F	GTACCAGCATTTAGCCCACTATG
UBR2-R	TGCAAGAATATGTAGGCTCTCCT
STUB1-F	AGCAGGGCAATCGTCTGTTC
STUB1-R	CAAGGCCCGGTTGGTGTAATA
FBXL12-F	CACTATGCCCAAGCTCAGAGT
FBXL12-R	GACGATGACCATACAGTGGGG
TRIM40-F	ACATCTCTTCTGTCGAGTGTGC
TRIM40-R	GGCAGATATAGCCTGTCCCTA
USP7-F	GGAAGCGGGAGATACAGATGA
USP7-R	AAGGACCGACTCACTCAGTCT
PJA2-F	TGTTGCAGTACATCACAGTGAG
PJA2-R	AGCACTACAAGCTCCTGGAATA
ALG13-F	TTTGACGACCTCATTGCGTGT
ALG13-R	TAAGTCGGTTGTAACCAAGGC
NIX-F	ATGTCGTCCCACCTAGTCGAG
NIX-R	TGAGGATGGTACGTGTTCCAG
TOLLIP-F	TGGGCCGACTGAACATCAC
TOLLIP-R	GTGGATGACCTTATTCCAGCG
TAX1BP1-F	AAGAAACAGCACAACTTCGAGA

Oligonucleotides	Target
TAX1BP1-R	TGGATGTAGCATCACTGAACCT
FUNDC1-F	CCTCCCCAAGACTATGAAAGTGA
FUNDC1-R	AAACACTCGATTCCACCACTG
FAM134B-F	CTCAGCCACTGTATTGCAGAA
FAM134B-R	AACAATGGACACAAAAATGCACA
NCOA4-F	GCTCAGCAGCTCTACTCGTTA
NCOA4-R	GGCACACAGAGACTTGATTGG
