# Supplementary material for: Targeting the Lysosomal Degradation of Rab22a‐NeoF1 Fusion Protein for Osteosarcoma Lung Metastasis
Source: Adv Sci (Weinh). 2022 Dec 18;10(5):2205483. doi: 10.1002/advs.202205483 (PMC9929137; doi:10.1002/advs.202205483)
Supplement: Supplementary file 3 — Supplemental Table 2 [file ADVS-10-2205483-s001.pdf]

ADVANCED SCIENCE
Open Access

Supporting Information

for *Adv. Sci.*, DOI 10.1002/advs.202205483

Targeting the Lysosomal Degradation of Rab22a-NeoF1 Fusion Protein for Osteosarcoma Lung Metastasis

Cuiling Zeng, Li Zhong, Wenqiang Liu, Yu Zhang, Xinhao Yu, Xin Wang, Ruhua Zhang, Tiebang Kang and Dan Liao**

Table 2

SiRNA	Target
TRIM40_001	CCACAGAAATTAGAGGTTA
TRIM40_002	CGGCCAGAATCCTTGACAT
TRIM40_003	GGACGGCCAAGGAATTAGA
FBXL12_001	CCAAGCTCAGAGTCCTTGA
FBXL12_002	TCCGAGATGTGCGCAAGAT
FBXL12_003	TGCAGGAGCTCAGCTATCT
USP7_001	GAATGACATGTACGATGAA
USP7_002	GAGCGACCTTACCCAAGTT
USP7_003	TAAGGACCCTGCAAATTAT
PJA2_001	GCAGGAGGGTATCAGACAA
PJA2_002	GAACAATGCTGTCCAATCT
PJA2_003	GTTAGATTCTGTACCATTA
UBR2_001	GGCGTAGGTCTGTTCGATA
UBR2_002	GGTTCACACCTATGAACAA
UBR2_003	GCCACTCAAAGTTCAAGTT
STUB1_001	CTGTGAAGGCGCACTTCTT
STUB1_002	GCTCTTCGAATCGCGAAGA
STUB1_003	AGCGCTGGAACAGCATTGA
ALG13_001	GGTCATCTCTTCTATTGTA
ALG13_002	GGAACTGGCAAAGCAGCTA
ALG13_003	GGGTCATCTCTTCTATTGT
PINK1_001	GGACGCTGTTCCTCGTTAT
PINK1_002	CCAACAGGCTCACAGAGAA
PINK1_003	GGCTGGTGATCGCAGATTT
FLT3_001	GGACTCGGATCAAATCTCA
FLT3_002	GAAGGCATCTACACCATTA
FLT3_003	GAATGTGCTTACATTTGAA
EGFR_001	GAGGAAATATGTACTACGA
EGFR_002	GGAGCGAATTCCTTTGGAA
EGFR_003	GCAGAGGAATTATGATCTT
P62_001	GGAGTCGGATAACTGTTCA
P62_002	TGAGGAAGATCGCCTTGGA
P62_003	GGACCCATCTGTCTTCAAA
OPTN_001	GGCTTACCTTGTTCAAAGA
OPTN _002	GAGCTTGTTTATACTAACA
OPTN _003	CCAAGAATTACTTCGAACA
NBR1_001	GGAGTGGATTTACCAGTTA
NBR1 _002	GGTGCAGTATCATAGTAGA
NBR1 _003	GAGCCCTGATAACATTGAA
NDP52_001	GAGCAAGTATTCCTTTCCA
NDP52 _002	GAAGCAGAATGAAACTACT

NDP52 _003	GCAGATTGCTCAGTTACAT
C-CBL_001	CTACCAGCATCTCCGTACT
C-CBL _002	GAGCTTTCGACAGGCTCTA
C-CBL _003	TCGGATTACTAAAGCAGAT
BNIP3L_001	GAATCAGGACAGAGTAGTT
BNIP3L_002	GGAAAAGTGGAGCCATGAA
BNIP3L_003	CAGACACCCTAAACGTTCT
STBD1_001	GCCGGAGCACTTTTCGTTT
STBD1_002	GGAGCTGGTCACCAAACCA
STBD1_003	TCCGGGAGGCCATCAGAGT
NIX_001	GAATCAGGACAGAGTAGTT
NIX_002	GGAAAAGTGGAGCCATGAA
TOLLIP_001	GCATGATCAACCTCGTCAT
TOLLIP_002	TGGTCCTGATGCCAACAGT
TOLLIP_003	GCGTGGACTCTTTCTATCT
TAX1BP1_001	CCTCTACTGTAGATGTAAA
TAX1BP1_002	CAGTGATGCTGTCAACGTA
TAX1BP1_003	GGAACACGAACTAAGAAGA
FUNDC1_001	GCAGCACCTGAAATCAACA
FUNDC1_002	CAAGCAGAACATTGTGATA
FUNDC1_003	GTAGCTACCCAGATTGTAA
FAM134B_001	GCAGAATCATGGATGAATT
FAM134B_002	CCACTGAGCTCAAGAGAAA
FAM134B_003	TCAGCATATTTCTTCAAGA
NCOA4_001	AGATGAGATGGACCTATCA
NCOA4_002	GGCTCATGCTAGTTCAGCA
