# Supplementary material for: Targeting the Lysosomal Degradation of Rab22a‐NeoF1 Fusion Protein for Osteosarcoma Lung Metastasis
Source: Adv Sci (Weinh). 2022 Dec 18;10(5):2205483. doi: 10.1002/advs.202205483 (PMC9929137; doi:10.1002/advs.202205483)
Supplement: Supplementary file 4 — Supplemental Table 3 [file ADVS-10-2205483-s006.pdf]

ADVANCED SCIENCE
Open Access

Supporting Information

for *Adv. Sci.*, DOI 10.1002/advs.202205483

Targeting the Lysosomal Degradation of Rab22a-NeoF1 Fusion Protein for Osteosarcoma Lung Metastasis

Cuiling Zeng, Li Zhong, Wenqiang Liu, Yu Zhang, Xinhao Yu, Xin Wang, Ruhua Zhang, Tiebang Kang and Dan Liao**

Table 3

Oligonucleotides	Target
STUB1 Sg1	CCGTTCCCCAGCTTACAGCC
STUB1 Sg2	TGGCCGTGTATTACACCAAC
STUB1 Sh1	CTGTGAAGGCGCACTTCTT
STUB1 Sh2	AGCGCTGGAACAGCATTGA
SgNDP52	GGAAGAAGCTCGAGCAGACA
PINK1 Sg1	CTGGGGCAATGTAGGCATGG
PINK1 Sg2	GAAATCCGACAACATCCTTG
lentiCRISPR V2-F	tcttGTGGAAAGGACGAAACACCg
lentiCRISPR V2-R142	cctagctagcgaattcAAAAAAgcac
